# Data‐ and Theory‐Guided Design of Dual‐Role V‐Doped RuO_2_ for High‐Performance Acidic Oxygen Evolution

**DOI:** 10.1002/anie.8887957

**Published:** 2026-06-07

**Authors:** Zhongliang Liu, Heng Liu, Kai Zhou, Miaomiao Liu, Tianrui Xue, Jian Zhang, Yiting Song, Jialin Cui, Hao Li, Huihui Li, Chunzhong Li

**Affiliations:** ^1^ Key Laboratory for Ultrafine Materials of Ministry of Education School of Chemical Engineering East China University of Science and Technology Shanghai China; ^2^ Advanced Institute for Materials Research (WPI‐AIMR) Tohoku University Sendai Japan; ^3^ Key Laboratory of Interfacial Physics and Technology Shanghai Institute of Applied Physics Chinese Academy of Sciences Shanghai People's Republic of China; ^4^ Shanghai Engineering Research Center of Hierarchical Nanomaterials School of Materials Science and Engineering East China University of Science and Technology Shanghai China

**Keywords:** deprotonation, Lewis acids, oxygen evolution reaction, theory‐guided

## Abstract

Developing efficient acidic oxygen evolution reaction (OER) catalysts is crucial for proton exchange membrane water electrolyzers (PEMWE). By mining a dataset of 718 reported catalysts, we statistically identified that multi‐metal Ru‐based oxides significantly outperform monometallic counterparts (median overpotential: 210 vs. 283 mV). Guided by this insight, microkinetic modeling screened 20 metal dopants, pinpointing vanadium as a promising candidate. The synthesized V‐doped RuO_2_ (RV) exhibits an ultralow overpotential of 193 ± 1 mV at 10 mA cm^−2^ and robust stability for 3000 h. In a practical PEMWE device, RV achieves an industrial current density of 1 A cm^−2^ at only 1.725 V and sustains operation for 140 h at 200 mA cm^−2^. Mechanistic studies reveal that V‐doping plays a dual role in RuO_2_. It induces Lewis acidic Ru sites to accelerate deprotonation kinetics, while simultaneously acting as a dynamic redox buffer to prevent Ru over‐oxidation. This work shows how data‐ and theory‐guided screening, combined with mechanistic investigation, can accelerate the discovery and understanding of high‐performance RuO_2_‐based acidic OER catalysts.

## Graphical abstract

A data‐ and theory‐guided paradigm, leveraging large‐scale data mining of 718 catalysts and microkinetic modeling, identifies V‐doped RuO_2_ as optimal for acidic OER. Vanadium doping drives electron withdrawal from Ru centers, generating Lewis acidic sites that polarize O–H bonds and accelerate deprotonation kinetics. Experimental validation achieves an ultralow overpotential of 193 ± 1 mV at 10 mA cm^−2^.

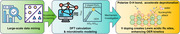

## 1 Introduction

Proton exchange membrane water electrolyzers (PEMWE) offer a promising pathway for sustainable hydrogen production, yet their efficiency is heavily constrained by the anodic acidic oxygen evolution reaction (OER) [1, 2, 3]. However, the acidic OER is kinetically sluggish and thermodynamically demanding, requiring highly efficient electrocatalysts to reduce the overpotential and improve energy efficiency [4, 5, 6]. While ruthenium (Ru) is recognized for its superior intrinsic activity [7, 8, 9, 10] compared to iridium (Ir), optimizing Ru‐based structures to maximize energy efficiency requires navigating a vast multidimensional space [4, 11, 12]. Traditional trial‐and‐error approaches are often inefficient in identifying promising active sites among the infinite combinatorial possibilities [4, 5, 13]. Therefore, integrating historical experimental data with theory‐guided screening provides a practical strategy to narrow down the search space and accelerate the discovery of improved acidic OER catalysts.

**FIGURE 1:**
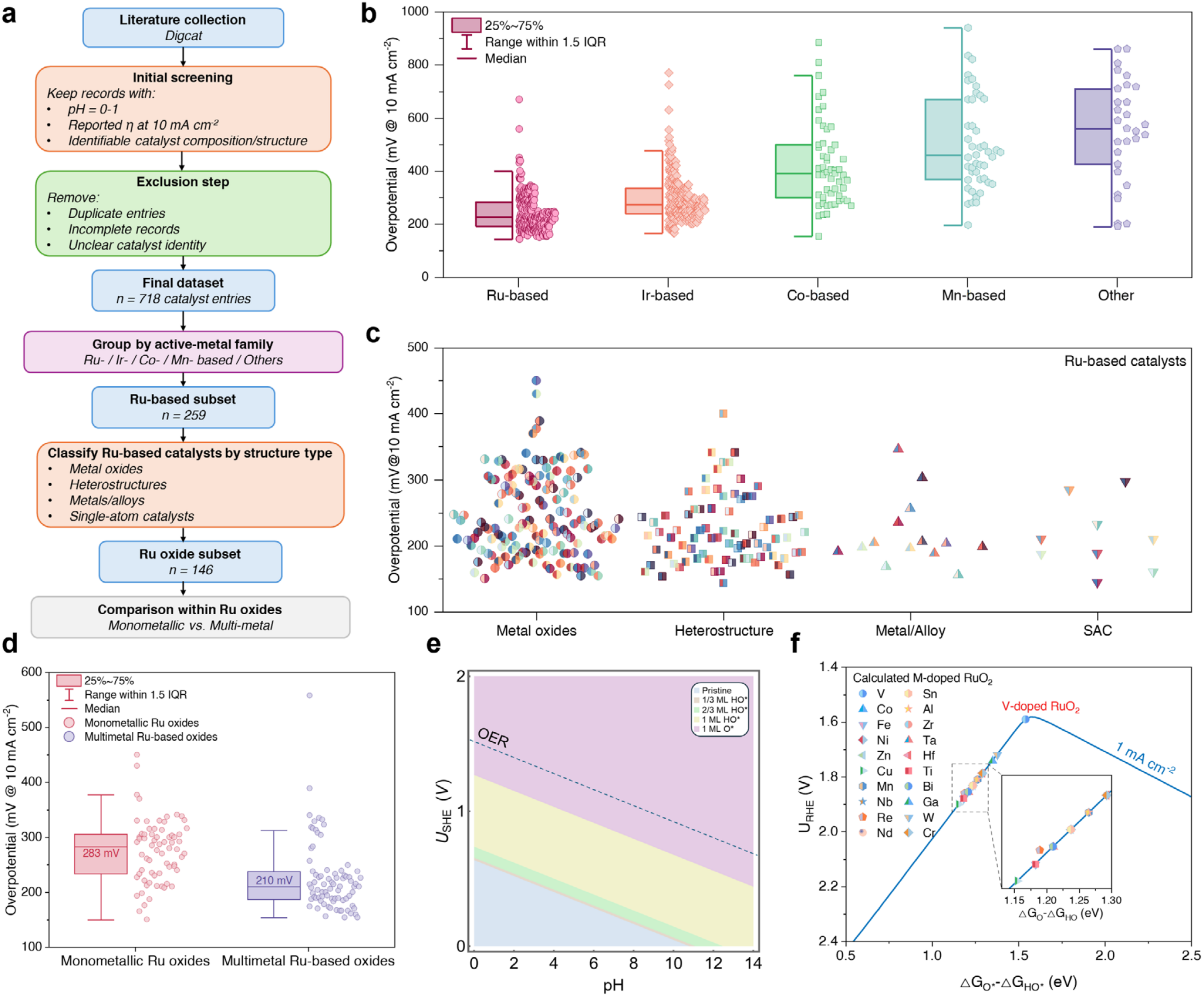
Large‐scale data mining and theoretical analysis of acidic OER catalysts. (a) Workflow of literature screening, data classification, and statistical analysis for the data‐mining study. (b) Statistical distribution (presented as hybrid box‐and‐scatter plots) of overpotentials at 10 mA cm^−2^ in acidic media (pH 0–1), classified by the active metal element (Ru, Ir, Co, Mn, and others). (c) Scatter plots of overpotentials for Ru‐based catalysts categorized by structure type: metal oxides (monometallic or multi‐metal oxides), heterostructures (including supported, core‐shell, and other heterojunctions), metals/alloys, and single‐atom catalysts (SAC). (d) Comparison of OER activity between monometallic Ru oxides and multimetal Ru‐based oxides (at least one secondary metal). All data are also available in the public DigCat database: https://www.digcat.org/. (e) Calculated 2D surface Pourbaix diagram as a function of potential (vs. U_SHE_) and pH (temperature = 298.15 K). Different color regions indicate the most energetically favorable surface state corresponding to specific pH values and potential (vs. SHE). The dashed line delineates the region where the OER occurs. (f) Microkinetic volcano activity model with the predicted performance of M‐doped RuO_2_ (M = V, Co, Fe, Ni, Zn, Cu, Mn, Nb, Re, Nd, Sn, Al, Zr, Ta, Hf, Ti, Bi, Ga, W, and Cr), building on the surface state of 1 ML O*.

To identify a suitable material class for acidic OER, we conducted a large‐scale data mining analysis of 718 catalysts reported over the past decade (the screening workflow and detailed statistics are summarized in Figure 1a and Table S1). Statistical analysis of the overpotential distribution at 10 mA cm^−2^ (pH 0–1) reveals that Ru‐based catalysts exhibit the lowest median overpotential compared to Ir‐, Co‐, and Mn‐based systems as depicted in Figure 1b (all data were also uploaded to the public *DigCat* database: https://www.digcat.org/) [14]. To further refine our search, we categorized the Ru‐based catalysts by structure type. As shown in Figure 1c and Figure S1, the dataset is dominated by metal oxides (146 data points) and heterostructures (90 data points), whereas metals/alloys (14 data points) and single‐atom catalysts (9 data points) are much less frequently reported. Among these, Ru‐based metal oxides demonstrate consistently high performance. A deeper statistical comparison within this oxide subset (Figure 1d) reveals an important trend: multi‐metal Ru‐based oxides containing at least one secondary metal exhibit a median overpotential of 210 mV, which is significantly superior to the 283 mV median of monometallic Ru oxides. This statistical evidence strongly suggests that heteroatom doping is a promising strategy to break the activity limits of pure RuO_2_. However, the aggregated experimental evidence does not unambiguously reveal which dopant delivers the largest enhancement, underscoring the need for theory‐guided screening.

Bridging this gap requires moving from statistical trends to atomic predictive insight. Unlike conventional thermodynamic descriptors, which often overlook the complexity of surface coverage under operating conditions, we employed advanced microkinetic modeling coupled with surface Pourbaix diagrams [12, 15]. With the rational assumption that minor doping would not change the surface states [12], this approach provides a reasonable guide for identifying the thermodynamically favored surface state of metal‐doped RuO_2_ under acidic OER conditions, which is covered by ∼1 monolayer (ML) O* (Figure 1e). Building upon this surface state, microkinetic modeling was implemented to construct the OER volcano plot [16], with which 20 distinct metal dopants on the RuO_2_ (110) surface (M‐doped RuO_2_, where M = V, Co, Fe, Ni, etc.) were systematically screened (Figure 1f and Table S2). Our theoretical predictions identify vanadium (V) as a particularly promising dopant that positions the catalyst near the *Sabatier* optimum of the activity volcano, suggesting that V‐doping can favorably modulate the binding energies of key oxygenated intermediates. To experimentally corroborate the theoretical screening, several M‐doped RuO_2_ catalysts (RuM, M = Ga, Cr, Bi, and Ti) were synthesized under the same preparation conditions and evaluated for OER activity. The measured activity trend is generally consistent with the DFT predictions (Figure S2), providing additional experimental support for the reliability of the theoretical screening rather than serving as a rigorous cross‐dopant validation of the theoretical model.

Guided by these data‐driven and theoretical insights, we synthesized the predicted V‐doped RuO_2_ catalyst (RV) via a sol‐gel method [17, 18] and further investigated its dual role in promoting deprotonation kinetics and stabilizing Ru under acidic OER conditions. Electrochemical measurements show that RV exhibits an ultralow overpotential of 193 ± 1 mV at 10 mA cm^−2^ and remarkable stability (3000 h at 10 mA cm^−2^), significantly outperforming commercial RuO_2_. Furthermore, we combine in situ spectroscopy and multiple electrochemical analyses to elucidate that V‐doping induces electron withdrawal from Ru centers, creating high‐valence Lewis acidic Ru sites that effectively polarize surface O–H bonds. This electronic modulation accelerates the deprotonation kinetics of key intermediates, thereby minimizing the energy barrier for the OER. At the same time, V also serves as a multivalent redox buffer that mitigates Ru over‐oxidation and helps preserve catalyst integrity under acidic OER conditions. Together, these results show that integrating data mining, theory, and experiment can help narrow the dopant search space while uncovering mechanistic design principles for RuO_2_‐based acidic OER catalysts.

## 2 Results and Discussion

### 2.1 Catalyst Synthesis and Structural Characterization

**FIGURE 2:**
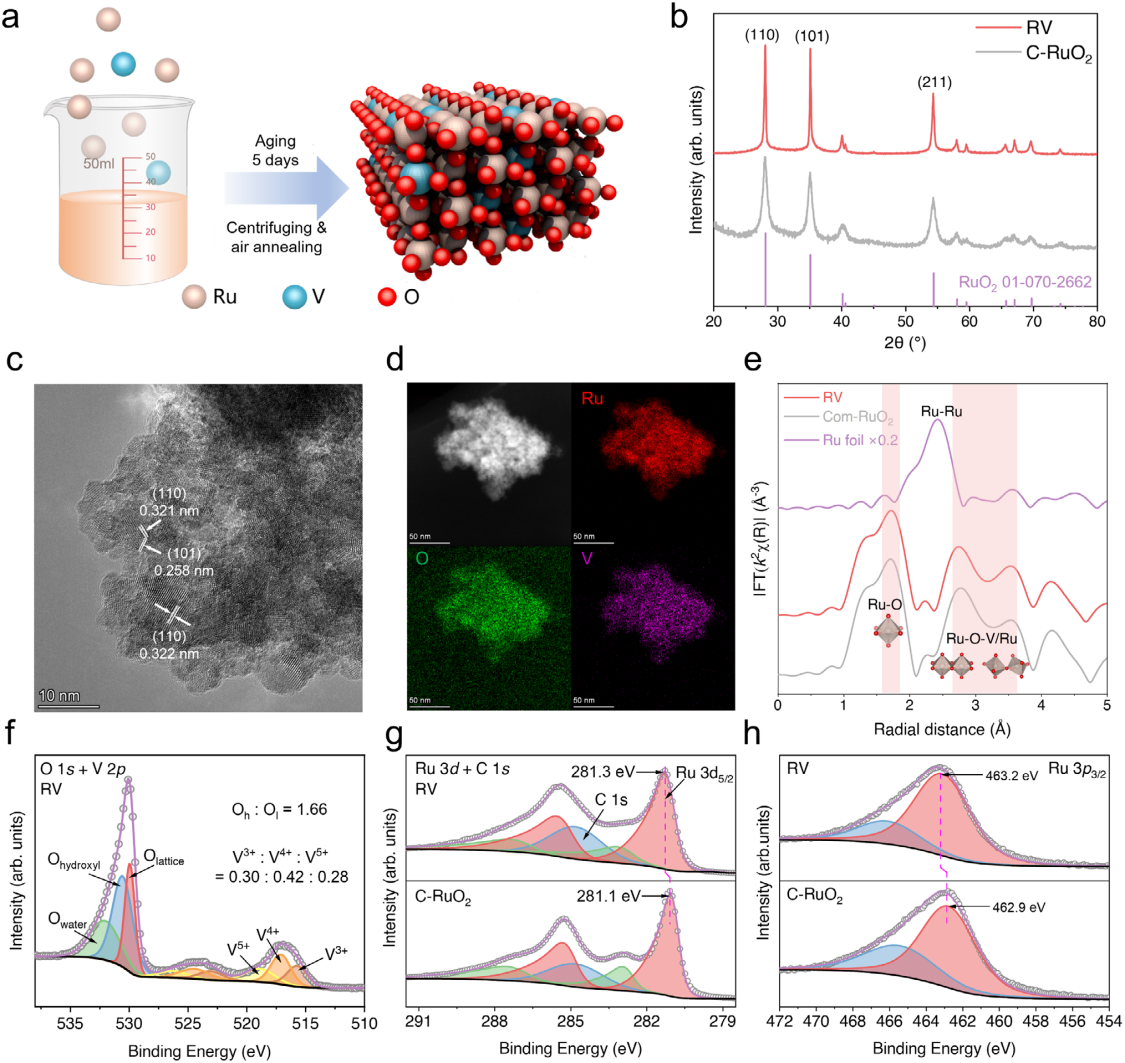
Catalyst synthesis and structural characterization. (a) Schematic of the synthesis procedure. (b) XRD patterns of RV and C‐RuO_2_ catalysts. (c) HRTEM image and (d) HAADF‐STEM image and the corresponding elemental mapping of RV. (e) FT‐EXAFS spectra at the Ru K‐edge of RV and C‐RuO_2_ catalysts. (f) O 1*s* + V 2*p* XPS spectrum of RV. (g) Ru 3*d* + C 1*s* and (h) Ru 3*p*_3/2_ XPS spectra of RV and C‐RuO_2_ catalyst.

Guided by the theoretical screening results, which identified vanadium as a promising dopant, we aimed to experimentally synthesize the V‐doped RuO_2_ (denoted as RV) to validate its acidic OER performance. The catalyst was prepared via a facile sol‐gel method followed by air annealing (Figure 2a) [12]. This synthetic strategy enables the homogeneous dispersion of metal precursors during the 5‐day aging period, after which air annealing oxidizes and crystallizes the gel to yield a uniformly V‐doped rutile RuO_2_ catalyst. To achieve optimal activity, the V content and annealing conditions were rigorously pre‐screened and optimized to 25 at.% and 400°C for 1 h, respectively, based on the overpotential at 10 mA cm^−2^ derived from linear sweep voltammetry (LSV) (Figure S3).

The crystallographic structure and elemental composition of the RV catalyst were first thoroughly characterized using a combination of X‐ray diffraction (XRD), X‐ray photoelectron spectroscopy (XPS), and electron microscopy. XRD patterns (Figure 2b) show three principal reflections indexed to the rutile RuO_2_ (110), (101), and (211) planes, consistent with commercial rutile RuO_2_ (denoted C‐RuO_2_) and the standard reference (PDF#01‐070‐2662). High‐resolution transmission electron microscopy (HRTEM, Figure 2c) further reveals a nanoparticle morphology with lattice spacings of 0.321 nm and 0.258 nm, corresponding to the rutile (110) and (101) planes, respectively. High‐angle annular dark‐field scanning transmission electron microscopy (HAADF‐STEM) combined with energy‐dispersive x‐ray spectroscopy (EDS) elemental mapping shows a uniform distribution of Ru, V, and O throughout the nanoparticles. The V content in RV was determined from the XPS survey, and the Ru:V atomic ratio is estimated to be approximately 2:1 (Table S3). To atomically resolve the influence of V doping, Fourier‐transformed extended x‐ray absorption fine structures (FT‐EXAFS) were employed to analyze the first‐ and second‐shell coordination around the Ru centers. As shown in Figure 2e, both RV and C‐RuO_2_ display three main peaks at essentially the same radial distances. The first peak arises from the first‐shell Ru–O coordination within a single [RuO_6_] octahedron, whereas the second and third peaks originate from two distinct connectivity modes between adjacent [RuO_6_] and/or [VO_6_] octahedra. These features strongly indicate the substitutional incorporation of V into the rutile RuO_2_ lattice, resulting in the formation of Ru–O–V motifs without disrupting the host structure.

Given that V is a typical multivalent metal with a stable +5 oxidation state [19, 20], its incorporation is expected to modulate the electronic structure of the RuO_2_ framework through strong Lewis acidity [21]. This modulation was investigated via XPS. As depicted in Figure 2f, simultaneous deconvolution was employed due to the partial overlap of the O 1*s* and V 2*p* regions. The O 1*s* spectrum resolves into three distinct peaks: lattice oxygen (O_l_, 529.9 eV), adsorbed hydroxyl species (O_h_, 530.6 eV), and adsorbed water (O_w_, 532.2 eV) [22]. Notably, RV exhibits a significantly higher O_h_:O_l_ ratio (1.66) compared to C‐RuO_2_ (1.25, Figure S4). This increase is attributed to the enhanced Lewis acidity of the V‐modified surface, which facilitates water polarization and subsequent deprotonation. The V 2*p* spectrum is deconvoluted into three spin‐split doublets (Figure 2f). The V 2*p*_3/2_ peaks centered at 515.7, 517.1, and 518.9 eV are assigned to V^3+^, V^4+^, and V^5+^ species, respectively [23, 24]. With a determined composition ratio of 0.30:0.42:0.28 (V^3+^:V^4+^:V^5+^), these results confirm the coexistence of mixed valence states of V incorporated into the RuO_2_ lattice. As displayed in Figure 2g,h, the Ru 3*d* and 3*p* spectra reveal that the binding energies of Ru 3*d*_5/2_ (281.3 eV) and Ru 3*p*_3/2_ (463.2 eV) in RV are positively shifted relative to C‐RuO_2_ (281.1 and 462.9 eV). These shifts indicate an increase in the Ru valence state, driven by the electron‐withdrawing effect of the Lewis acidic V dopants through Ru‐O‐V motifs. This interaction depletes the electron density around Ru, enhancing its Lewis acidity. Such electron redistribution is intrinsically favorable for the OER because the resulting electron‐deficient Ru sites are more susceptible to nucleophilic attack by H_2_O. Concurrently, the enhanced Lewis acidity simultaneously polarizes and weakens the O–H bonds of adsorbed intermediates, thereby facilitating deprotonation kinetics.

### 2.2 Acidic OER Performance

**FIGURE 3:**
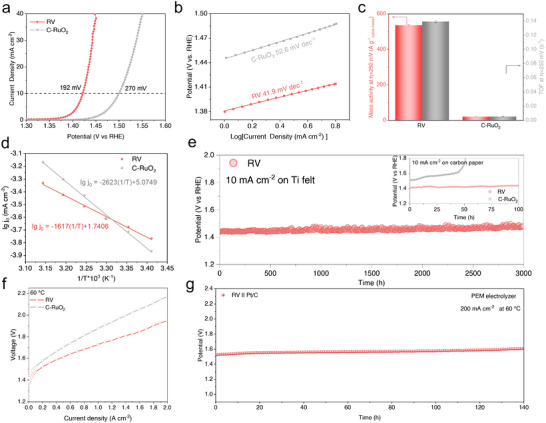
Electrochemical OER performance of RV and C‐RuO_2_ catalysts. (a) *i*R‐corrected LSV curves with error bars (*n* = 3). (b) Tafel plots and corresponding slopes. (c) Comparison of mass activity and TOF at 1.48 V versus RHE (*n* = 3). (d) Linear fitting of Arrhenius plots. (e) Chronopotentiometric stability of RV loaded on Ti felt at 10 mA cm^−2^ in a three‐electrode configuration (inset shows the stability comparison on carbon paper). (f) Polarization curves of the assembled PEMWE cell and (g) the corresponding stability test of RV at 200 mA cm^−2^ and 60°C.

The electrochemical OER performance of RV and C‐RuO_2_ catalysts was evaluated using a three‐electrode configuration in 0.1 M HClO_4_. The setup employed a catalyst‐loaded rotating disk electrode (RDE), a Pt mesh, and an Ag/AgCl electrode as the working, counter, and reference electrodes, respectively. The catalytic activity was initially assessed via LSV polarization curves (Figure 3a). Notably, RV requires an ultra‐low overpotential of only 193 ± 1 mV to reach 10 mA cm^−2^, substantially outperforming C‐RuO_2_ (271 ± 5 mV). The accelerated reaction kinetics of RV are further corroborated by Tafel analysis, which yields a reduced Tafel slope of 41.9 mV dec^−1^ compared to 52.6 mV dec^−1^ for C‐RuO_2_. Additionally, Nyquist plots derived from EIS measurements at 1.40 V versus RHE (Figure S5) reveal a smaller semicircle diameter for RV, indicating a significantly lower charge transfer resistance (R_ct_). Collectively, these metrics confirm that V doping effectively promotes OER kinetics, establishing RV as a promising catalyst for acidic OER.

To understand the origin of this high apparent activity, the electrochemically active surface area (ECSA) was estimated via double‐layer capacitance (C_dl_), determined from CV scans in the non‐Faradaic region (Figure S6a,b). As shown in Figure S6c, the linear fit yields a *C*_dl_ of 43.2 mF cm^−2^ for RV, which is approximately 6.5 times larger than that of C‐RuO_2_. This substantial increase implies a greater abundance of exposed active sites on the RV surface. Beyond the active surface area effect, the ECSA‐normalized LSV curves (Figure S7) reveal that RV exhibits superior specific activity. The O_2_ faradaic efficiency (FE_O2_) was measured by water displacement, and the FE_O2_ is approximately 99.2% across different current densities (Figure S8). Based on this, and assuming that all loaded Ru atoms are catalytically active, RV achieves a mass activity of 536.3 ± 6.0 A g_Ru_^−1^ and an estimated lower‐bound turnover frequency (TOF) of 0.139 ± 0.002 s^−1^, representing a dramatic improvement over C‐RuO_2_ (19.7 ± 1.2 A g_Ru_^−1^ and 0.0052 ± 0.0003 s^−1^, respectively) (Figure 3c and Table S4). These results indicate that the superior apparent activity of RV arises from the combined contributions of a substantially increased density of accessible active sites and enhanced intrinsic activity per site, both of which contribute to its high acidic OER performance.

To elucidate the intrinsic kinetic origin of this performance, in situ EIS measurements were conducted to probe charge transfer dynamics at the catalyst‐electrolyte interface. Nyquist plots for RV and C‐RuO_2_ (Figure S9a,b) reveal that the *R*_ct_ for both catalysts decreases monotonically with increasing potential (Figure S9c). Notably, RV consistently exhibits lower *R*_ct_ values than C‐RuO_2_ across the entire potential range, indicating a significantly reduced energy barrier for interfacial electron transfer. The interfacial dynamics were further analyzed via Bode phase plots (Figure S10a,b) and the potential‐dependent evolution of the phase angle (Figure S10c). As the potential approaches the OER onset, the phase angle peak shifts toward higher frequencies, reflecting a reduced characteristic time constant (τ) and thus an increased reaction rate [25]. Crucially, the faster phase angle response in RV suggests that V doping facilitates the rapid turnover of key oxygenated intermediates (e.g., *OH and *OOH) [15, 26, 27]. These enhanced kinetics are further corroborated by the apparent activation energy (*E*_a_) derived from temperature‐dependent LSV (Figure S11). As shown in Figure 3d, the smaller Arrhenius slope for RV indicates a lower activation energy compared to C‐RuO_2_, confirming a reduced kinetic barrier induced by V doping. Collectively, these results indicate that the V‐induced Lewis acidity accelerates charge transfer and facilitates oxygenated intermediates deprotonation at Ru active sites, thereby enhancing intrinsic OER kinetics.

Moving beyond fundamental kinetics, we evaluated the industrial feasibility and practical viability of the RV catalyst by assessing its stability and performance in both three‐electrode and PEMWE configurations. Figure 3e highlights the robust long‐term durability of RV. When loaded on Ti felt, the RV catalyst sustains stable electrolysis for an impressive 3000 h at 10 mA cm^−2^. To benchmark this against commercial standards, a comparison on carbon paper substrates is provided in the inset. In stark contrast to C‐RuO_2_, which deactivates within 50 h, RV maintains robust performance throughout the 100‐h test without apparent deterioration. We further assembled a PEMWE to evaluate the electrocatalytic performance of RV in a practical device. As shown in the polarization curves (Figure 3f), RV reaches the industrial current density of 1 A cm^−2^ at only 1.725 V (60°C), which is significantly lower than the 1.874 V required by C‐RuO_2_. This reduced voltage translates to an energy cost for RV calculated to be 45.94 kWh/kg H_2_, representing a significant saving of 3.83 kWh/kg H_2_ compared to C‐RuO_2_ (49.77 kWh/kg H_2_). Furthermore, RV demonstrated high stability in the PEMWE, operating at 200 mA cm^−2^ for 140 h with negligible degradation. Taken together, these results identify RV as a promising acidic OER catalyst, with outstanding half‐cell durability and encouraging PEMWE performance. The benchmarking in Table S5 places RV among competitive recent Ru‐based systems, while differences in PEMWE current density, operating temperature, and test duration should be considered when comparing device‐level stability.

### 2.3 Strong Deprotonation Capacity Drives Highly Active OER

To elucidate the mechanism underlying the enhanced deprotonation capacity and resultant rapid kinetics, we first sought to identify the dominant OER pathway on RV. To this end, TMA^+^ probe and pH‐dependence experiments were performed to assess the extent of lattice oxygen participation (LOM). TMA^+^ cations selectively interact with peroxo‐like *O_2_2^−^ intermediates characteristic of LOM [28, 29], while a pronounced pH‐dependence of OER activity on the RHE scale is widely regarded as a feature of non‐concerted proton–electron transfer associated with LOM [30, 31]. Compared with C‐RuO_2_ (*ρ* = 0.697), RV exhibits a weaker pH dependence (*ρ* = 0.317) and a smaller TMA^+^‐induced activity suppression (Figures S12–S13), indicating that V doping suppresses lattice oxygen participation and drives the reaction toward a conventional adsorbate evolution mechanism (AEM). This conclusion is consistent with the AEM pathway adopted in our microkinetic model. Having established the AEM‐dominated pathway, we then employed multiple characterization techniques, including square‐wave voltammetry (SWV), pulse voltammetry (PV), molecular probe‐assisted CV, and in situ Fourier transform infrared spectroscopy (FTIR), to investigate the deprotonation kinetics of key oxygenated intermediates within this framework.

**FIGURE 4:**
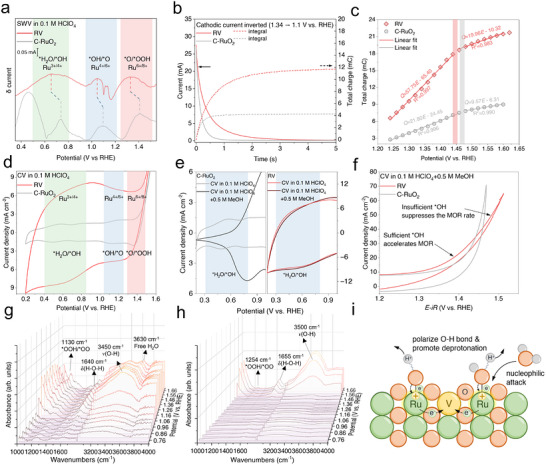
Mechanism investigation of V‐induced Lewis acidity for enhanced deprotonation capacity. (a) SWV profiles, (b) selected cathodic current response (1.34‐1.1 V vs. RHE), and the corresponding charge integration. (c) Linear relationship between total charge and potential, (d) CV curves in 0.1 M HClO_4_. Comparison of CV scans in 0.1 M HClO_4_ with 0.5 M MeOH in the (e) non‐Faradaic and (f) Faradaic regions of RV and C‐RuO_2_ catalysts. In situ FTIR spectra of (g) RV and (h) C‐RuO_2_. (i) Schematic of the proposed mechanism for performance enhancement.

Initially, SWV was utilized to probe the specific redox transitions of surface active sites, leveraging its high sensitivity to exclude non‐faradaic charging currents (Figure 4a) [32]. The distinct redox peaks observed at approximately 0.7, 1.1, and 1.4 V versus RHE are assigned to the Ru^3+/4+^, Ru^4+/6+^, and Ru^6+/8+^ transitions, respectively. These redox processes are coupled with the stepwise deprotonation of intermediates: *H_2_O → *OH, *OH → *O, and *O → *OOH. Compared to C‐RuO_2_, the redox peaks of RV exhibit a distinct negative shift, signifying that the oxidation of surface Ru sites becomes thermodynamically more favorable. This facile generation of high‐valence, Lewis acidic Ru sites effectively polarizes O–H bonds, thereby promoting the subsequent deprotonation process.

Following the identification of favorable high‐valence Ru site formation, we further investigated the deprotonation behavior during the OER catalytic region using PV to quantify the accumulated oxidative charge on the catalyst surface, which serves as a descriptor for intrinsic OER activity [33, 34]. The charge is determined by integrating the cathodic current during a quick potential step, representing the total charge required to reduce the high‐valent active species accumulated at the anodic potential back to their base state. This charge is primarily stored as pseudocapacitance, reflecting Ru redox transitions coupled with intermediate deprotonation (Figures S14–S16). As shown in Figure 4b, RV exhibits a significantly larger integrated cathodic charge than C‐RuO_2_, indicating a superior capacity for oxidative charge storage. The potential‐dependent charge profiles (Figure 4c) further reveal that RV possesses a substantially higher pseudocapacitance (57.75 mF) in the pre‐catalytic region compared to C‐RuO_2_ (21.80 mF). These results confirm that V doping enhances the capability to accumulate high‐valence Ru species and deprotonated intermediates at lower potentials.

Notably, both catalysts exhibit a bilinear behavior consistent with observations for other noble metal oxides [33, 34]. This transition is consistent with a shift in the rate‐determining step (RDS) from the initial *OH deprotonation (*OH → *O + H^+^ + e^−^) toward the subsequent water nucleophilic attack (*O + H_2_O → *OOH + H^+^ + e^−^) at higher potentials. Crucially, the inflection point for RV occurs at a potential ∼0.2 V lower than that of C‐RuO_2_. This shift suggests that the energy barrier for the preceding *OH deprotonation step is significantly reduced on the RV surface. Consequently, the facilitated deprotonation enables the rapid accumulation of active *O intermediates, triggering the subsequent nucleophilic attack at lower overpotentials and driving the superior OER kinetics.

To further investigate the subsequent deprotonation of *OOH, H_2_O_2_ was utilized as a molecular probe, given that H_2_O_2_ oxidation proceeds via a *OOH intermediate analogous to the OER mechanism (*+H_2_O_2_ → *OOH + H^+^ + e^−^, *OOH → *+O_2_ + H^+^ + e^−^) [35]. As depicted in Figure S17, RV exhibits a reduced overpotential for H_2_O_2_ oxidation compared to C‐RuO_2_, supporting the accelerated formation and deprotonation of *OOH intermediates to release O_2_.

Having established that V doping facilitates the formation of high‐valence Ru species and accelerates the deprotonation of oxygenated intermediates (*OH → *O, *O → *OOH, *OOH → O_2_), we further employed methanol as a molecular probe to elucidate the kinetics of the initial water nucleophilic attack (*+H_2_O → *OH + H^+^ + e^−^) [36]. First, baseline CVs were recorded in 0.1 M HClO_4_ (Figure 4d). RV exhibits a markedly higher capacitive current around the *H_2_O/*OH transition potential compared to C‐RuO_2_, signifying a greater abundance of surface *OH intermediates. Upon introducing 0.5 M methanol, C‐RuO_2_ suffers from a dramatic loss of capacitive current below 0.7 V versus RHE (Figure 4e, left), attributed to the competitive adsorption of methanol, which poisons the Ru sites and blocks water attack. In stark contrast, the CV profile of RV remains robust and nearly identical to its baseline in the presence of methanol (Figure 4e, right). This suggests that the water nucleophilic attack on RV is kinetically superior, allowing water to outcompete methanol for surface sites and rapidly generate *OH species. Furthermore, the methanol oxidation reaction (MOR) on RV initially exhibits enhanced activity due to its dependence on surface *OH species (Figure 4f), yet the current subsequently decreases at higher potentials. This suppression is consistent with fast kinetics, in which surface *OH species are preferentially deprotonated to *O rather than participating in methanol oxidation, leading to a depletion of *OH intermediates.

While multimodal electrochemical characterizations strongly suggest that V doping promotes the formation of high‐valence Lewis‐acidic Ru sites and boosts consecutive deprotonation steps, direct in situ spectroscopic observation is indispensable for molecular‐level validation. To bridge this gap, in situ FTIR was performed to monitor the evolution of surface intermediates under operating conditions. Figure 4g,h display the potential‐dependent infrared spectra for RV and C‐RuO_2_, respectively. As the potential sweeps anodically from 0.736 to 1.696 V, a distinct absorption band emerges at approximately 1130 cm^−1^ for RV (1254 cm^−1^ for C‐RuO_2_), which is characteristic of the linearly bonded superoxo intermediate (*O–O or *O–OH) [15, 37, 38]. Additionally, peaks observed around 1650 cm^−1^ and 3500 cm^−1^ are assigned to the bending mode [*δ*(H–O–H)] of adsorbed water molecules and the stretching mode [*ν*(O–H)] of adsorbed *OH, respectively [15]. Comparing the spectra of the two catalysts reveals a significant difference in the onset potential for intermediate formation. For RV (Figure 4g), the characteristic *OH and *OO/*OOH bands become discernible at a potential as low as 1.096 and 1.276 V, respectively. Conversely, on C‐RuO_2_ (Figure 4h), these species only appear at much higher potentials of 1.456 and 1.516 V. This earlier appearance on RV supports that V doping significantly lowers the energy barrier for deprotonation, thereby facilitating the formation of key intermediates. Crucially, a close inspection of the vibrational frequencies reveals a redshift for the intermediates on RV compared to C‐RuO_2_. Specifically, the *δ*(H–O–H) mode of adsorbed water and *ν*(O–H) mode of adsorbed *OH on RV are located at 1640 and 3450 cm^−1^, respectively, which are lower than those observed for C‐RuO_2_ (1655 and 3500 cm^−1^). Based on the harmonic oscillator model [39], the observed redshift indicates a reduced force constant and, consequently, a weakened O–H bond. Notably, a new peak corresponding to free water appears at 3630 cm^−1^ on RV at elevated potentials (> 1.516 V vs. RHE) [38]. This suggests a disruption of the rigid hydrogen bond network, facilitating rapid water transport at the electrode–electrolyte interface, a key factor for high‐current‐density performance [27]. These in situ spectroscopic insights and electrochemical analyses are consistent with our proposed mechanism: V doping induces electron withdrawal, creating Lewis acidic Ru sites. These sites promote nucleophilic water attack and accelerate deprotonation by effectively polarizing surface O–H bonds (Figure 4i).

### 2.4 Multivalent V Dynamically Stabilizes Ru Active Sites

While the modulation of Lewis acidity and electronic structure by V doping significantly enhances the intrinsic OER activity, the long‐term durability of Ru‐based catalysts remains a formidable challenge. This instability stems primarily from the thermodynamic tendency of active Ru species toward over‐oxidation and dissolution (forming soluble RuO_4_) under high anodic potentials [40, 41]. Therefore, sustaining the active phase without structural collapse is critical. Herein, we propose that the multivalent nature of V serves as an “electronic redox buffer,” which dynamically stabilizes the Ru active sites through a self‐adjusting redox mechanism during the OER process.

To elucidate the structural origin of this superior stability, the post‐catalytic morphology and composition of the RV catalyst were first examined. After 24 h of chronopotentiometric operation (RV‐24 h), HRTEM and EDS mapping reveal that the catalyst preserves its original rutile crystal structure and uniform elemental distribution without noticeable phase segregation or collapse (Figure S18).

**FIGURE 5:**
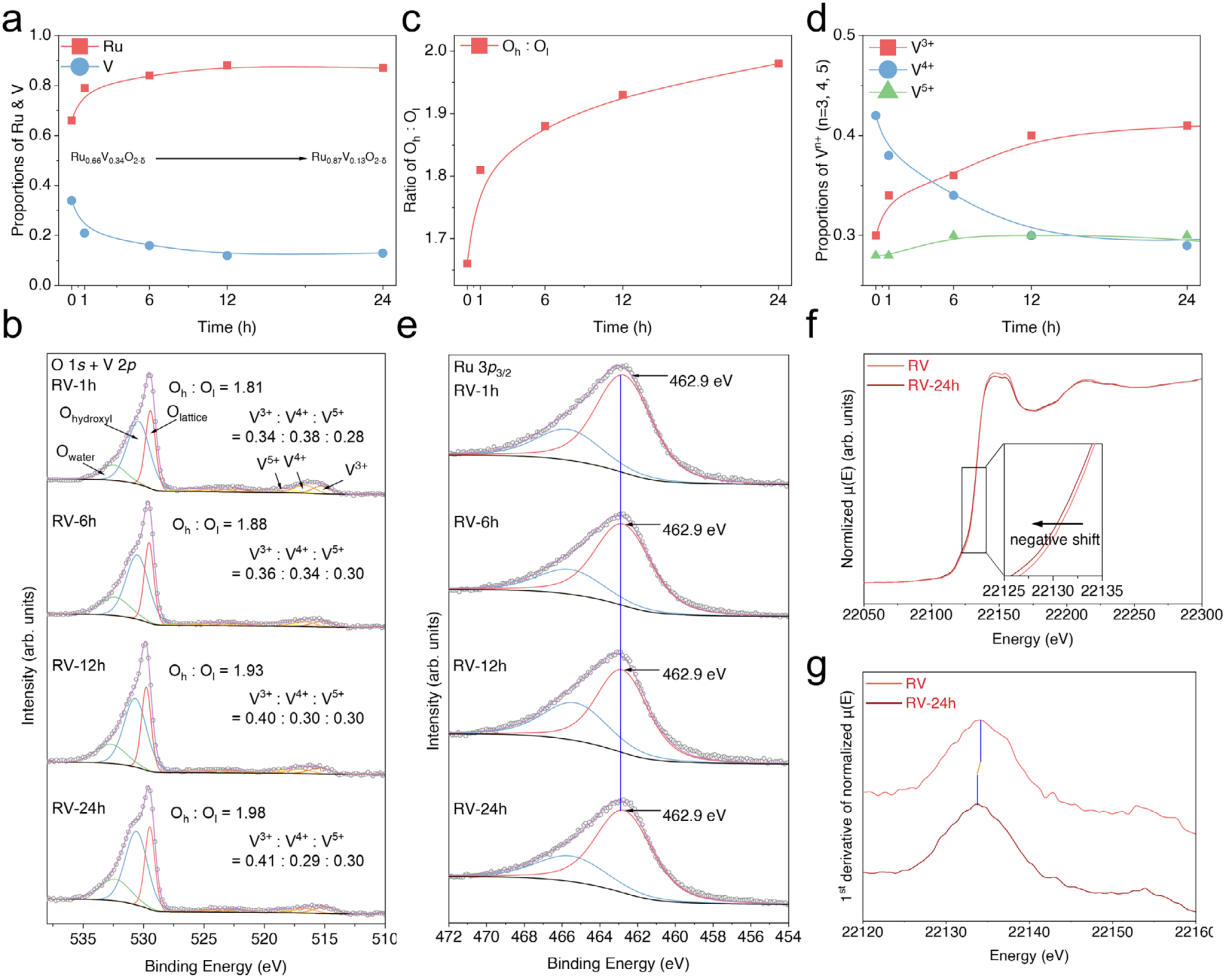
Dynamic stabilization of Ru active sites. (a) Evolution of the Ru/V atomic ratio in RV as a function of stability testing time, determined by XPS. (b) O 1*s* + V 2*p* XPS spectra of RV recorded at different stability testing intervals (1, 6, 12, 24 h). (c) Calculated ratio of O_h_:O_l_ derived from XPS results. (d) Relative proportions of V^3+^, V^4+^, and V^5+^ species over 1, 6, 12, 24 h of OER. (e) Ru 3*p*_3/2_ XPS spectra of RV at 1, 6, 12, 24 h of OER. (f) Ru K‐edge XANES spectra of the RV and RV‐24 h samples. (g) First derivative of the normalized μ(E) spectra in (f).

To probe the surface chemical evolution at the atomic level, ex situ XPS analyses were performed on RV catalysts collected at different reaction stages (1, 6, 12, and 24 h, denoted as RV‐1 h to RV‐24 h). Quantitative analysis indicates that the surface atomic ratio of Ru:V increases from an initial ∼2:1 to 6.7:1 (Figure 5a and Table S6). This trend suggests the partial leaching of thermodynamically unstable surface V species, leading to a surface composition reconstruction that likely triggers electronic rearrangement. Further deconvolution of the spectra provides deeper insights into this dynamic restructuring (Figure 5b). In the O 1*s* XPS spectra, the ratio of O_h_:O_l_ increases progressively with reaction time (Figure 5c). This confirms that the RV surface continuously accumulates oxidative charge by deprotonating water molecules to form stable surface hydroxyls, thereby driving the OER.

More importantly, a distinct evolution in V valence states is concurrently observed. The proportion of metastable V^4+^ (3*d* ^1^) species decreases, while the populations of stable V^3+^ (3*d*2) and V^5+^ (3*d* ^0^, empty orbital) species increase (Figure 5d). This valence redistribution is attributed to the disproportionation of unstable V^4+^ to achieve more stable electronic configurations (V^3+^ and V^5+^). Building upon the previous finding that V doping increases the Ru valence state through Ru‐O‐V motifs, we propose that this dynamic electron transfer among multivalent V species is mediated toward the Ru sites through the Ru‐O‐V motifs. This self‐adjusting process is in line with partial buffering of the electronic fluctuations of the Ru centers, dynamically stabilizing their electronic structure and inhibiting the irreversible over‐oxidation of high‐valent Ru species into soluble forms.

Indeed, ex situ Ru 3*p*_3/2_ XPS spectra (Figure 5e) confirm that the average valence state of Ru decreases to a level comparable to C‐RuO_2_ after just 1 h of reaction and remains stable without further oxidation throughout the 24‐h test (denoted as RV‐24 h). This stabilization effect is further corroborated by ex situ XANES spectra (Figure 5f) and its first derivative (Figure 5g), which display a negative shift of the absorption edge in the RV‐24 h sample compared to the pristine RV sample. Electrochemical CV analysis further supports different over‐oxidation tendencies. C‐RuO_2_ displays a distinct reduction current peak assigned to the Ru^6+^/Ru^8+^ redox transition between 1.3 and 1.4 V versus RHE (Figure S19), indicating that C‐RuO_2_ might partially over‐oxidize at higher potentials. Crucially, this reduction peak is absent in RV (Figure S19). Furthermore, this over‐oxidation peak in C‐RuO_2_ gradually increases and becomes more apparent during prolonged OER operation (30, 60, 90, 120 min), while RV exhibits reduced current density without an obvious peak identification (Figure S20). To further substantiate the suppression of Ru dissolution, inductively coupled plasma‐mass spectroscopy (ICP‐MS) analysis was performed to quantify the dissolved Ru concentration in the electrolyte at different reaction intervals at 10 mA cm^−2^ (1, 2, 4, 8, and 24 h). As shown in Figure S21, the Ru dissolution from RV remains consistently lower than that from C‐RuO_2_ throughout the entire testing period, confirming that V doping effectively mitigates Ru leaching under acidic OER conditions. Taken together, the ex situ characterizations and electrochemical analysis support that the multivalent V doping dynamically stabilizes the Ru active sites, effectively preventing over‐oxidation during the OER process and thereby ensuring superior long‐time durability.

## 3 Conclusion

In summary, by integrating large‐scale data mining of 718 reported catalysts with high‐accuracy microkinetic volcano modeling, we recognized V as a promising dopant for improving RuO_2_ toward acidic OER. The resulting V‐doped RuO_2_ experimentally validates this data‐driven catalyst discovery strategy, delivering an exceptional ultra‐low overpotential of 193 ± 1 mV at 10 mA cm^−2^ and sustaining stable operation for over 3000 h under harsh acidic conditions. Mechanistic studies further support that the performance enhancement originates from the dual role of V: it induces the formation of high‐valence Lewis acidic Ru sites that accelerate the rate‐determining deprotonation step, while simultaneously serving as a redox buffer to maintain the integrity of the active sites. Beyond identifying a high‐performance electrocatalyst candidate for PEM water electrolysis, this work establishes V doping as an effective route to simultaneously enhance the activity and stability of RuO_2_ and offers mechanistic insight into how dopant chemistry can be leveraged to regulate acidic OER performance.

## Author Contributions

**Zhongliang Liu**: data curation, formal analysis, visualization, writing – original draft, writing – review and editing, methodology, validation, investigation. **Heng Liu**: data curation, formal analysis, methodology, visualization, writing – original draft, writing – review and editing, validation, investigation. **Kai Zhou**: data curation, formal analysis, investigation, methodology, validation. **Miaomiao Liu**: data curation, formal analysis. **Tianrui Xue**: data curation, formal analysis. **Jian Zhang**: data curation, formal analysis. **Yiting Song**: data curation, formal analysis. **Jialin Cui**: data curation, formal analysis. **Hao Li**: formal analysis, writing – original draft, supervision, conceptualization, visualization. **Huihui Li**: conceptualization, formal analysis, writing – original draft, visualization, investigation, supervision, funding acquisition, writing – review and editing. **Chunzhong Li**: conceptualization, formal analysis, visualization, writing – original draft, writing – review and editing, funding acquisition, investigation, supervision.

## Conflicts of Interest

The authors declare no conflicts of interest.

## Supporting information



The authors have cited additional references within the Supporting Information [42, 43, 44, 45, 46, 47, 48, 49, 50, 51].
**Supporting File**: anie73061‐sup‐0001‐SuppMat.docx.

## Data Availability

The data that support the findings of this study are available in the supplementary material of this article.
