# Supplementary material for: Data‐ and Theory‐Guided Design of Dual‐Role V‐Doped RuO2 for High‐Performance Acidic Oxygen Evolution
Source: Angew Chem Int Ed Engl. 2026 Jun 7;65(32):e8887957. doi: 10.1002/anie.8887957 (PMC13427137; doi:10.1002/anie.8887957)
Supplement: Supplementary file 1 — The authors have cited additional references within the Supporting Information [42, 43, 44, 45, 46, 47, 48, 49, 50, 51]. Supporting File: anie73061‐sup‐0001‐SuppMat.docx. [file ANIE-65-e8887957-s001.docx]

Supporting Information
©Wiley-VCH 2021
69451 Weinheim, Germany

**Data- and Theory-Guided Design of Dual-Role V-Doped RuO_2_ for High-Performance Acidic Oxygen Evolution**

Zhongliang Liu,^†[a]^ Heng Liu,^†[c]^ Kai Zhou,^[a]^ Miaomiao Liu,^[d]^ Tianrui Xue,^[a]^ Jian Zhang,^[a]^ Yiting Song,^[a]^ Jialin Cui,^[a]^ Hao Li,*^[c]^ Huihui Li,*^[a]^ and Chunzhong Li*^[a,b]^

**Abstract:** Developing efficient acidic oxygen evolution reaction (OER) catalysts is crucial for proton exchange membrane water electrolyzers (PEMWE). By mining a dataset of 718 reported catalysts, we statistically identified that multi-metal Ru-based oxides significantly outperform monometallic counterparts (median overpotential: 210 vs. 283 mV). Guided by this insight, microkinetic modeling screened 20 metal dopants, pinpointing vanadium as a promising candidate. The synthesized V-doped RuO_2_ (RV) exhibits an ultralow overpotential of 193±1 mV at 10 mA cm^-2^ and robust stability for 3000 hours. In a practical PEMWE device, RV achieves an industrial current density of 1 A cm^-2^ at only 1.725 V and sustains 140 hours at 200 mA cm^-2^. Mechanistic studies reveal that V-doping plays a dual role in RuO_2._ It induces Lewis acidic Ru sites to accelerate deprotonation kinetics, while simultaneously acting as a dynamic redox buffer to prevent Ru over-oxidation. This work shows how data- and theory-guided screening, combined with mechanistic investigation, can accelerate the discovery and understanding of high-performance RuO_2_-based acidic OER catalysts

Table of Contents

[Experimental Procedures 1](#_Toc223527594)

[Supplementary Figures, Tables and Notes 6](#_Toc223527595)

[References 24](#_Toc223527596)

[Author Contributions 24](#_Toc223527597)

Experimental Procedures

**Materials and chemicals**

Ruthenium chloride hydrate (RuCl_3_·xH_2_O, 99.9%), vanadium chloride (VCl_3_, 99.9%) and propylene oxide were purchased from Adams-beta. Acetone (C_3_H_6_O, AR) and anhydrous ethanol (C_2_H_6_O, ≥99.7%) were purchased from Sinopharm Chemical Reagent Co., Ltd. Perchloric acid (70.0~72.0%) and tetramethylammonium sulfate were purchased from Aladdin Scientific Corp. Nafion^®^ (5 wt. % in lower aliphatic alcohols and water, contains 15-20% water) was purchased from Sigma-Aldrich. Commercial ruthenium oxide (RuO_2_) was purchased from Premetek. Commercial Pt/C (20 wt. %) was purchased from Johnson Matthey. Carbon black (Vulcan XC-72) was purchased from Cabot. The YLS-30T gas diffusion layer (GDL) and Pt coated Ti felt were purchased from Sinero. All chemicals were used without any further purification.

**Synthesis of RV catalyst**

The R_3_V_1_O_x_ (RV) catalyst was prepared by a previously reported sol-gel method^[13]^ followed by air-annealing. Specifically, 0.3 mmol RuCl_3_·xH_2_O and 0.1 mmol VCl_3_ were dissolved in 2 mL ethanol. An ethanol‑water mixture (2 mL ethanol and 0.18 mL H_2_O) was prepared separately. Under an ice bath, the ethanol–water mixture and propylene oxide (1 mL) were added concurrently to the metal precursor solution under moderate stirring, and the resulting mixture was aged for 24 h to yield black precipitates. The supernatant was replaced with fresh acetone once daily for 5 days. The precipitates were then collected by centrifugation, washed with acetone three times, dried in a vacuum oven at 60°C for 2 h, and ground into a black powder. The powder was annealed in air at 400 °C for 1 h with a ramp rate of 5°C min^-1^ to yield the RV catalyst. To optimize the annealing conditions, a series of samples annealed at different temperatures (T) for 1 h were prepared and denoted as Ru_3_V_1_O_x_-T (T=300, 350, 400, 450, 500 °C). After identifying 400°C as the optimal annealing temperature, a set of Ru_y_V_1_O_x_ (y=2, 3, 4, 5) catalysts was synthesized to evaluate the effect of the Ru:V atomic ratio on catalytic performance.

**Characterization**

Transmission electron microscopy (TEM) and Scanning transmission electron microscopy (STEM) characterization were performed using ThermoFisher Talos F200X (FETEM, 200 kV). High angle annular dark field (HAADF)-STEM images were recorded using a convergence semi angle of 11 mrad, and inner- and outer collection angles of 59 and 200 mrad, respectively. Energy dispersive X-ray spectroscopy (EDS) was carried out using 4 in-column Super-X detectors. The X-ray diffraction patterns of as-prepared and used catalysts were obtained on an X-Ray Polycrystalline Diffractometer (D8 Advance, Bruker) with a LYNXEYE detector and Cu-Kα radiation source. The 2θ angle was scanned at a rate of 1.2° min^−1^ from 20° to 80°. The X-ray photoelectron spectroscopy (XPS) measurements and XPS depth profile were collected by the ThermoFisher Nexsa G2 surface analysis system with Al Kα radiation (1486.6 eV) under ultrahigh-vacuum conditions. The dissolution of metal ions in the electrolyte after the chronopotentiometry test was quantified by inductively coupled plasma-mass spectroscopy (ICP-MS, NexIon 2000, PerkinElmer). Synchrotron X-ray absorption spectroscopy spectra were collected at the BL14W1 beamline of the Shanghai Synchrotron Radiation Facility (SSRF). The corresponding data were analysed by Athena software (ver. 0.9.26).

**Electrochemical measurements**

The OER performance was evaluated in a three-electrode configuration using an electrochemical workstation (CHI760E, CH Instruments Inc.). An Ag/AgCl (3.5 M KCl) electrode served as the reference, and a Pt mesh was used as the counter electrode. The working electrode was prepared by drop-casting. Briefly, the catalyst (4.0 mg) and carbon black (1.0 mg) were dispersed in a mixed solvent of ethanol (980 μL) and Nafion solution (20 μL) to form an ink. After ultrasonication for 1 h in an ice bath, 15 μL of the ink was deposited onto a polished rotating disk electrode (RDE; diameter 5 mm, geometric area 0.196 cm^2^) and dried in air at room temperature, yielding a catalyst loading of ~0.3 mg cm^-2^.

To assess OER activity, linear sweep voltammetry (LSV) was recorded in 0.1 M HClO_4_ at a scan rate of 5 mV s^-1^ with the RDE rotating at 1600 r.p.m and without *i*R compensation during acquisition. Prior to LSV, the system was conditioned by cyclic voltammetry (CV) between 0.6 and 1.0 V vs Ag/AgCl for 10 cycles at 50 mV s^-1^. Post-measurement, 90% *i*R compensation was applied manually. The solution resistance for ohmic-drop correction was determined by electrochemical impedance spectroscopy (EIS) at 1.40 V vs RHE over 100 kHz–0.01 Hz with a 5 mV AC amplitude.

For durability testing, 4 mg of catalyst was dispersed in isopropanol (2 mL) and 5 wt. % Nafion solution (30 μL). The suspension was ultrasonicated for 1 h in an ice bath and spray-coated onto a gas diffusion layer (GDL; YLS-30T, 1 cm^2^) until a total mass loading of ca. 2.0 mg cm^-2^ was achieved. The OER stability test was conducted in an H-cell by chronopotentiometry at 10 mA cm^-2^ in 0.1 M HClO_4_, using Ag/AgCl (3.5 M KCl) and Pt mesh as the reference and counter electrodes, respectively.

All potentials are reported versus the reversible hydrogen electrode (RHE). Conversion followed E_RHE_=E_Ag/AgCl_+0.205 V+0.0591×pH. Values were corrected using 90% *i*R compensation as specified above.

**PEMWE test**

For practical PEMWE evaluation, the RV catalyst was dispersed in a water/isopropanol/PTFE mixture (catalyst: PTFE = 80:20 wt.%) and ultrasonicated for ≥1 h to obtain a uniform ink, which was subsequently drop-cast onto Pt-coated Ti felt. The coated anode was air-annealed at 350 °C for 3 h to remove residual PTFE, achieving a uniform loading of 2 mg cm^-2^. The cathode was fabricated by spraying a Pt/C ink containing 30 wt.% ionomer onto carbon paper, with the Pt loading controlled at 0.2 mg_Pt_ cm^-2^. A 1 cm^2^ PEM-WE cell employing a Nafion N115 membrane served as the proton conductor. The stack comprised Ti end plates, gaskets, the Nafion membrane, the cathode, and a second Ti end plate. Deionized water was supplied at 0.9 ml min^-1^. Polarization curves over 0.01–2.00 A cm^-2^ at 60 °C were collected via chronopotentiometry at stepped current densities. Durability was assessed by chronopotentiometry at 0.2 A cm^-2^ and 60 °C.

***In-situ* EIS measurements**

*In-situ* EIS was conducted using a DH7002 electrochemical workstation (Jiangsu Donghua Analytical Instruments Co., Ltd.) in 0.1 M HClO_4_ with a conventional three-electrode configuration. The working electrode was prepared by spray-coating following the same procedure used for the stability tests, with a catalyst loading of 0.5 mg cm^−2^. EIS measurements were performed at 1.23, 1.38, 1.43, 1.48, and 1.53 V vs. RHE to probe interfacial charge-transfer behavior at different stages of the OER. An AC amplitude of 5 mV was used, with frequencies spanning 100 kHz to 0.1 Hz. During measurements, the working electrode was held at each target potential for 60 seconds prior to data acquisition to minimize non-steady-state effects. The resulting Nyquist plots were fitted with an appropriate equivalent circuit to extract the charge-transfer resistance (R_ct_). *In-situ* EIS thus reveals the electronic transport characteristics of the catalyst under OER conditions, providing important insights into its electrochemical behavior during the reaction.

**Apparent activation energy measurements**

The electrochemical measurements were performed at various temperatures in 0.1 M HClO_4_ to determine the apparent activation energy of the acidic OER. The apparent activation energy *E*_a_ is related to the exchange current density *j*_0_ through the Arrhenius relationship (1):

$$\begin{aligned} j_{0}=Ae^{\frac{-E_{a}}{RT}}\#\left( 1 \right) \end{aligned}$$

Where A represents the apparent pre-exponential factor, R is the ideal gas constant (8.314 J K^-1^ mol^-1^), and T is the temperature in Kelvin (K). The value of E_a_ can be determined by performing a linear fit on the Arrhenius plot, using equation (2):

$$\begin{aligned} \left| \frac{\partial(lgj_{0})}{\partial\left( \frac{1}{T} \right)} \right|=-\frac{E_{a}}{2.303R}\#\left( 2 \right) \end{aligned}$$

The intercept of this linear fit corresponds to log A^[42]^.

**H_2_O_2_ redox experiments**

The working electrode with an RV catalyst loading of 0.5 mg cm^−2^ was initially conditioned by CV in 0.1 M HClO_4_ (50 mV s^−1^, 0.8–1.2 V vs RHE, 10 cycles) to stabilize the surface. Subsequently, the electrode was transferred to 0.1 M HClO_4_ containing 0.1 M H_2_O_2_, and CV was recorded over 0–1.23 V vs RHE for 3 cycles.

The reduction of H_2_O_2_ to H_2_O proceeds via the *OH intermediate, which is identical to the *OH species involved in the OER. The reduction steps are shown in equations (3) and (4). In contrast, the oxidation of H_2_O_2_ to O_2_ proceeds via the *OOH intermediate, as outlined in equations (5) and (6)^[27]^.

$$\begin{aligned} *+H_{2}O_{2}+H^{+}+e^{-}\to{}^{*}{OH}+H_{2}O\#\left( 3 \right) \end{aligned}$$

$$\begin{aligned} {}^{*}{OH}+H^{+}+e^{-}\to*+H_{2}O\#\left( 4 \right) \end{aligned}$$

$$\begin{aligned} *+H_{2}O_{2}\to{}^{*}{OOH}+H^{+}+e^{-}\#\left( 5 \right) \end{aligned}$$

$$\begin{aligned} {}^{*}{OOH}\to*+{O_{2}+H}^{+}+e^{-}\#\left( 6 \right) \end{aligned}$$

By analyzing the redox behavior of H_2_O_2_, one can compare the relative adsorption energies of the *OH and *OOH intermediates on the catalyst surface.

***In-situ* FTIR measurements**

*In-situ* Fourier transform infrared spectroscopy (FTIR) was performed using a designed electrochemical cell and an Infrared spectrometer (Bruker Vertex80) equipped with an MCT detector. Infrared reflection-absorption spectral mode was adopted, and a CaF_2_ crystal was used as the window where infrared light goes through. 40 µL of catalyst ink was dropped onto the glassy carbon electrode (with a diameter in 5 mm) to serve as the working electrode. The Pt wire and Ag/AgCl electrode were used as the counter and reference electrodes, respectively. *In-situ* FTIR spectra were recorded using stepped chronoamperometry method ranging from 0.736 V to 1.696 V (vs. RHE) in 0.06 V increments. The spectral acquisition was performed with a resolution of 2 cm^-1^ and 16 scans.

**Pulse voltammetry**

Pulse voltammetry (PV) was used to quantify charge accumulation on RV and C-RuO_2_ catalysts under acidic OER conditions. The working electrode was held at a constant low potential E_l_=1.10 V vs. RHE, stepped to a higher potential E_h_, for 5 s, and then returned to E_l_ for 5 s in a cyclic sequence. The E_h_ value increased from 1.22 to 1.60 V in 20 mV steps, while E_l_ was kept unchanged. For each step, the total accumulated charge was obtained by integrating the cathodic transient current over time, as the integrals of the cathodic pulses reflect the amount of oxidative charge stored in the electrocatalyst at the applied anodic potential^[25]^.

**Square-wave voltammetry**

Square-wave voltammetry (SWV) profiles were recorded using a step potential of 5 mV, a square-wave amplitude of 50 mV, and a frequency of 50 Hz in the potential range of 0.20–1.60 V vs. RHE. The differential (net) current (δ current) was plotted against the applied potential to effectively eliminate interference from background capacitive current.

**Methanol probe experiments**

To probe the formation and behaviour of surface *OH intermediates, methanol was introduced as a probe molecule into the electrolyte. All experiments were performed in a standard three-electrode cell using the same configuration as for the main electrochemical measurements. Initially, two CV cycles were recorded in 0.1 M HClO_4_ between 0.20 and 1.60 V vs. RHE at a scan rate of 50 mV s^-1^ to obtain the base voltammogram of the clean catalyst surface. Subsequently, the electrolyte was replaced with 0.1 M HClO_4_ containing 0.5 M methanol, and two further CV cycles were recorded in the potential range of 0.20–1.80 V vs. RHE at 50 mV s^-1^.

**ECSA measurement and calculation**

Double-layer capacitance (C_dl_) was evaluated by cyclic voltammetry (CV) within 0.6–0.8 V vs. RHE at scan rates of 20, 40, 60, 80, and 100 mV s^-1^ without electrode rotation. The anodic and cathodic currents at 0.7 V vs. RHE were plotted against scan rate, and the linear fit slope was taken as C_dl_. The electrochemical active surface area (ECSA) was estimated according to ECSA = C_dl_/C_s_, where C_s_ = 0.035 mF cm^-2^ was adopted as a commonly used specific capacitance value for noble-metal oxide electrodes. Because the actual specific capacitance can vary with surface composition, morphology, roughness, and oxide chemistry, the ECSA values reported here should be regarded as approximate estimates and are used primarily for relative comparison between samples rather than absolute quantification.

**Calculation of the mass activity**

The mass activity of the catalyst was calculated using equation (7):

$$\begin{aligned} j_{mass}=\frac{j_{geo}\times A}{m_{Ru}}\#\left( 7 \right) \end{aligned}$$

*m_Ru_* is the total Ru mass loaded onto rotating disk electrode, *A* is the geometric area of the electrode, and *j_geo_* is the geometric current density at 1.48 V vs. RHE after 90% *i*R compensation.

**Calculation of the turnover frequency (TOF)**

The TOF of the electrocatalysts was determined using equation (8):

$$\begin{aligned} TOF=\frac{j_{geo}\times A\times\eta}{4\times n\times F}\#\left( 8 \right) \end{aligned}$$

Where the *j_geo_* is the geometric current density at 1.48 V vs. RHE after 90% *i*R compensation, *A* is the geometric area of the electrode, *η* is the oxygen faradaic efficiency (assuming to be 100%), *F* is the Faraday constant, and *n* is the number of moles of active sites. Here, n was estimated by assuming that all loaded Ru atoms are catalytically active. The value was calculated based on the total mass loading and the molar mass of the catalyst derived from its specific composition. It should be noted that this assumption provides an upper-bound estimate of the active-site count; accordingly, the derived TOF values represent lower-bound estimates of the true per-site turnover frequency.

**Computational methods.**

Spin-polarized density functional theory (DFT) calculations were performed using the Vienna *ab initio* Simulation Package (VASP)^[43]^ with the projector augmented-wave (PAW) method^[44]^. A plane-wave basis set was employed to expand the Kohn–Sham wavefunctions^[45]^ describing the valence electrons, with a kinetic energy cutoff of 520 eV. Electron exchange–correlation effects were treated within the generalized gradient approximation (GGA)^[46]^ using the revised Perdew–Burke–Ernzerhof (RPBE) functional^[47]^. All atomic structures were fully relaxed until the residual forces on each atom were below 0.05 eV Å⁻¹. Brillouin zone integrations were carried out using a 2 × 3 × 1 Monkhorst–Pack *k*-point mesh. A vacuum layer of 15 Å was introduced along the surface to eliminate spurious interactions between periodic images. In slab models, the two bottommost layers were fixed at their bulk lattice positions, while all remaining layers were allowed to relax. The V/Ru ratio used in the doped model was based on the post-OER surface composition, which more reliably represents the actual working state. The Atomic Simulation Environment (ASE)^[48]^ was employed for structural manipulation, input file generation, and surface stability analysis. Computational parameters for bulk materials were adopted from the Materials Project database. Surface Pourbaix diagrams were constructed following the methodology proposed by Hansen *et al.*^[49]^, based on the computational hydrogen electrode (CHE) model developed by Nørskov *et al.*^[50]^, enabling evaluation of thermodynamic stability as a function of pH and electrode potential. The surface Pourbaix analysis was then used to define the thermodynamically favored OER surface for screening. This treatment is intended to capture the dominant surface state and dopant effect, while local reconstruction or V redistribution during operation may still occur. Zero-point energy and entropic contributions were incorporated under standard conditions (298.15 K) using corrections reported in previous studies. In addition, solvation corrections were applied to HO* intermediates to account for stabilization arising from hydrogen-bonding interactions, with correction values taken from the literature^[51]^. All computational structures generated in this work have been deposited in the ***DigCat*** platform ([www.digcat.org](http://www.digcat.org)).

Supplementary Figures, Tables and Notes


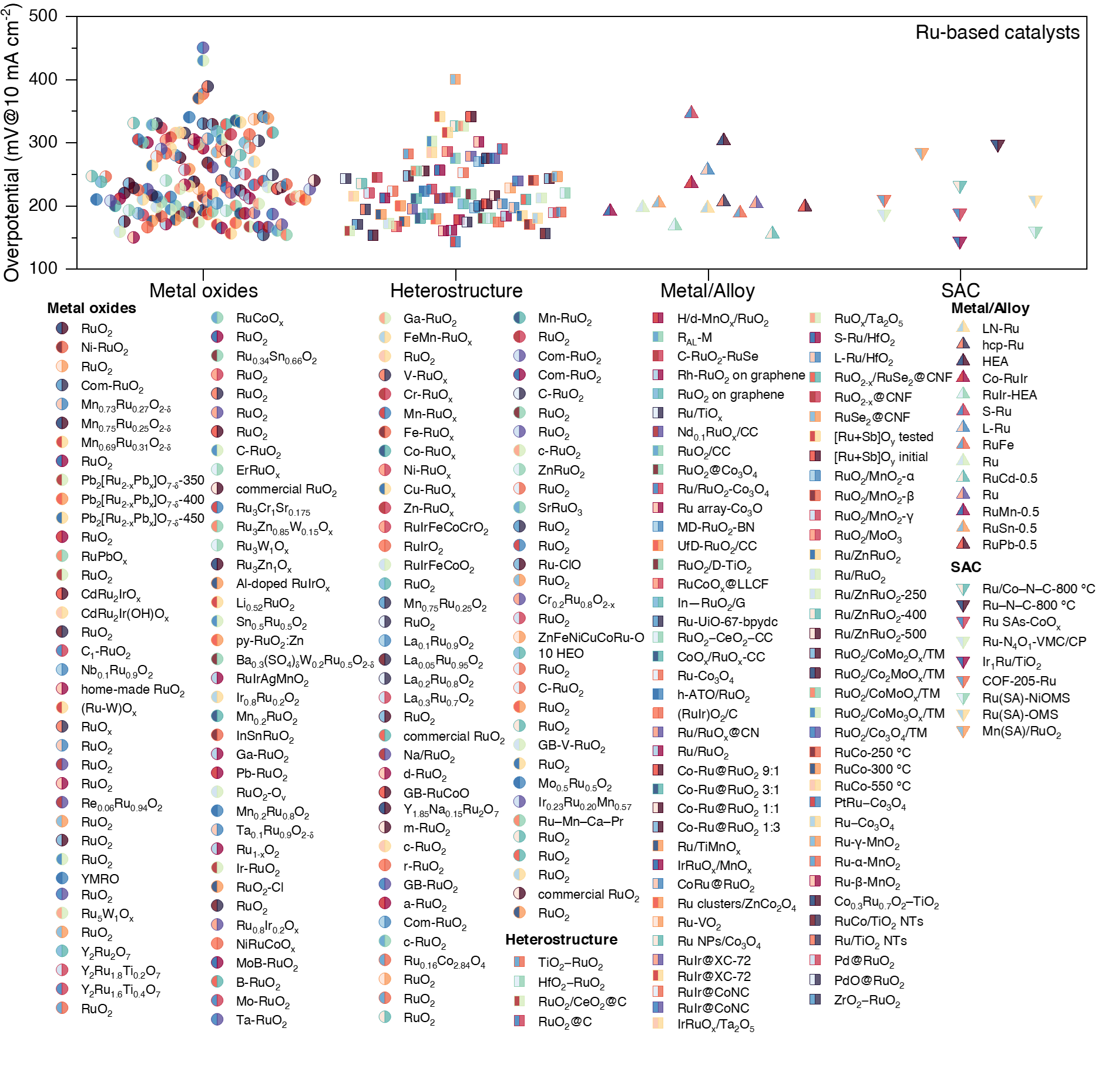


**Figure S1**. Scatter plots of overpotentials for Ru-based catalysts categorized by structure type with detailed catalysts information: metal oxides (monometallic or multi-metal oxides), heterostructures (including supported, core-shell, and other heterojunctions), metals/alloys, and single-atom catalysts (SAC).


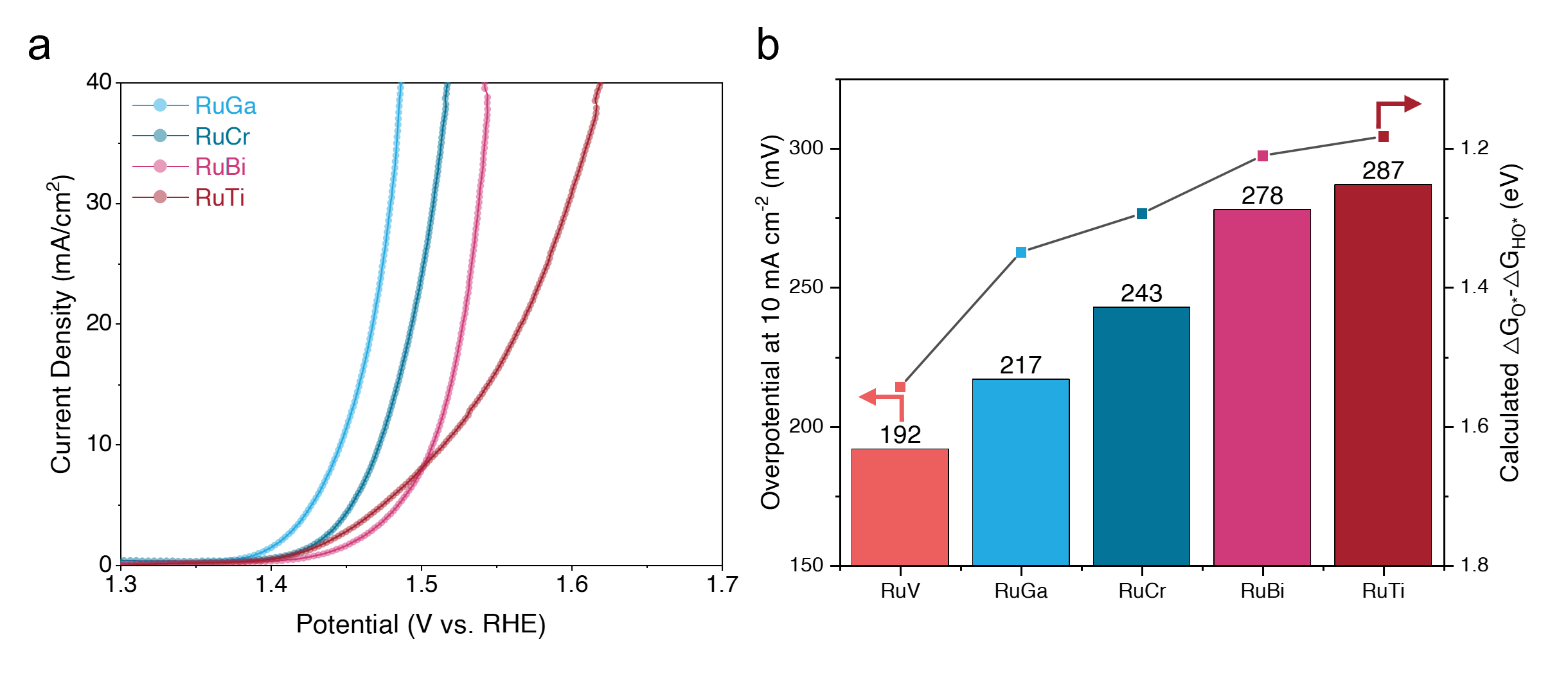


**Figure S2**. (a) LSV curves of synthesized RuGa, RuCr, RuBi, and RuTi. (b) Comparison of the experimental overpotentials at 10 mA cm^-2^ and the calculated descriptor values ΔG_O_-ΔG_HO_ for RuV, RuGa, RuCr, RuBi, and RuTi, showing a generally consistent trend between experiment and DFT prediction.


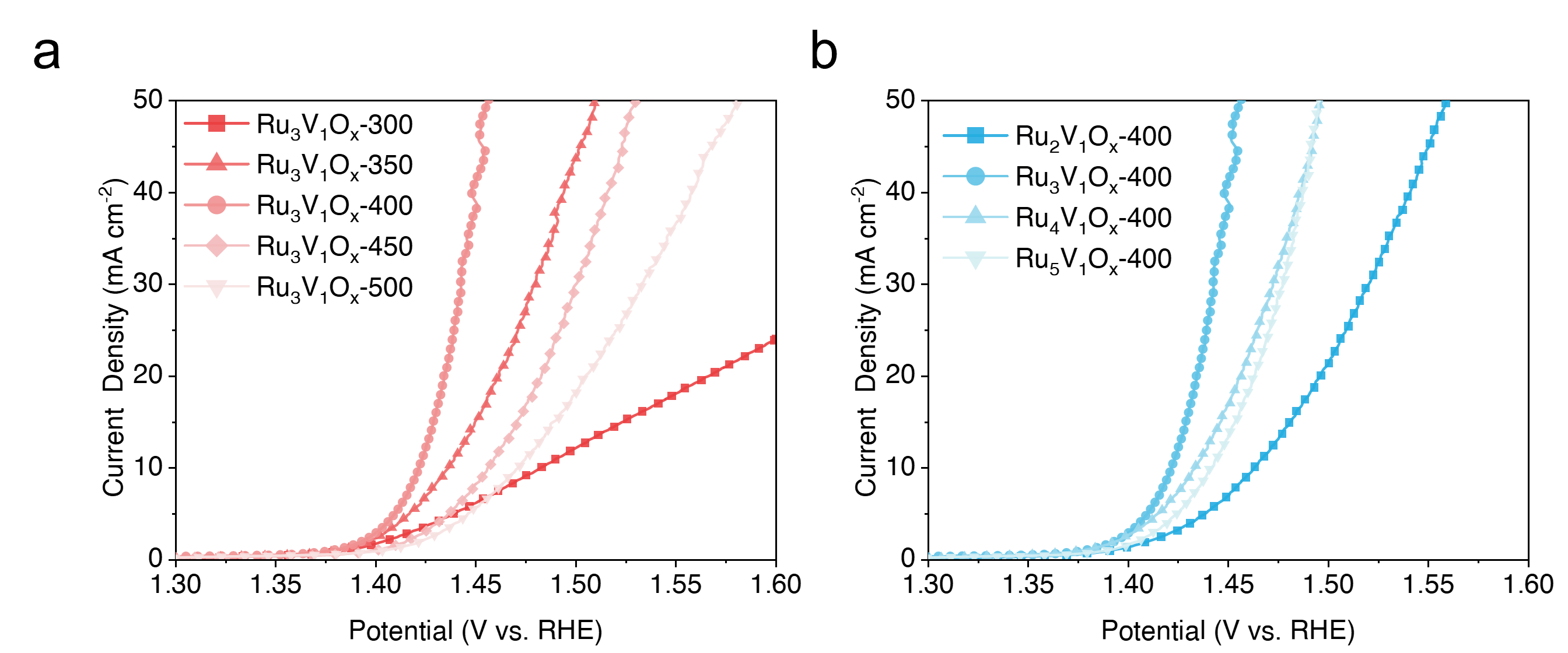


**Figure S3**. (a) LSV curves of Ru_3_V_1_O_x_-T (T=300, 350, 400, 450, 500 °C); T denotes the annealing temperature. (b) LSV curves of Ru_y_V_1_O_x_ annealed at 400 °C (y=2, 3, 4, 5); y denotes the Ru:V precursor ratio used during synthesis.


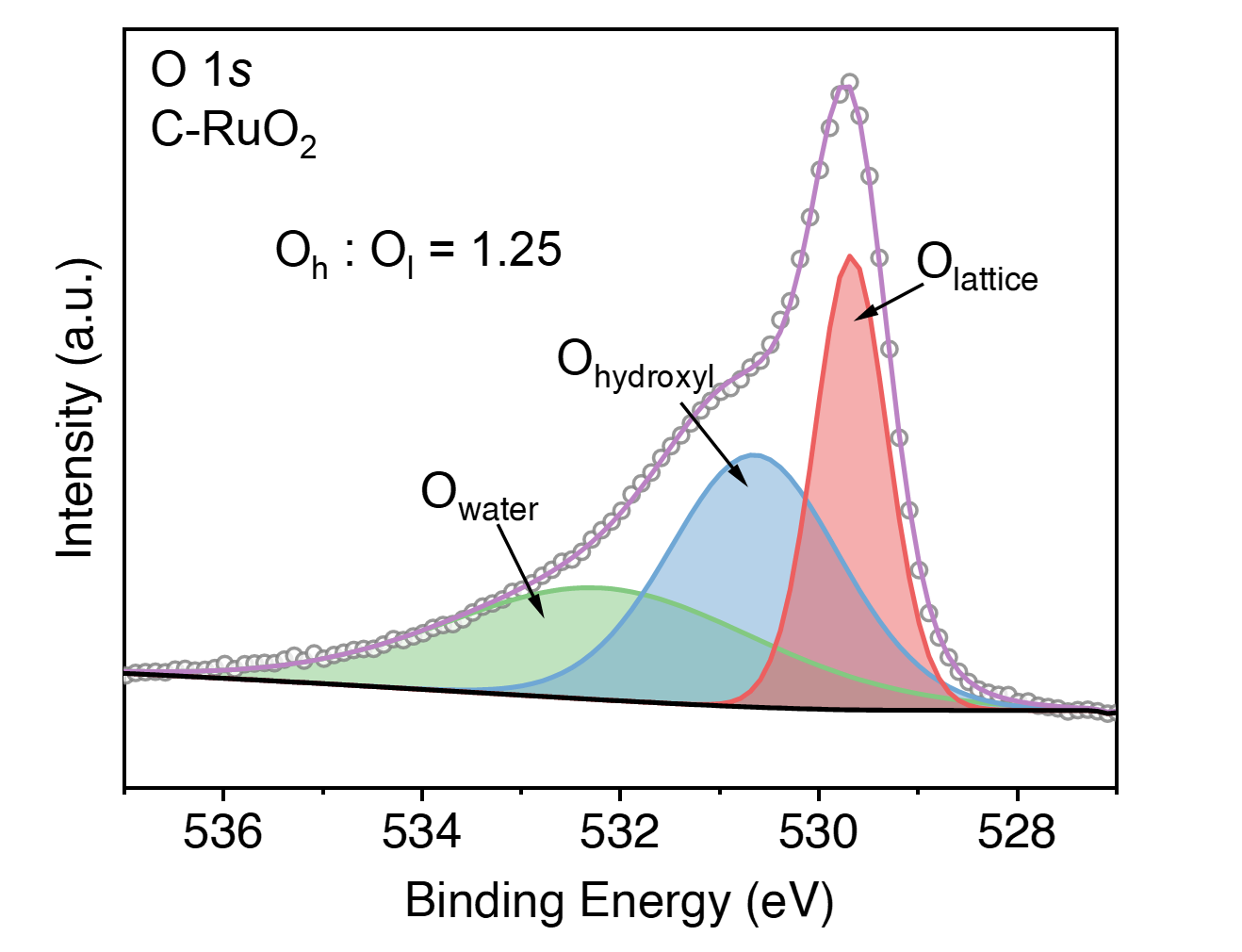


**Figure S4**. O 1*s* XPS spectra of the C-RuO_2_ catalyst.


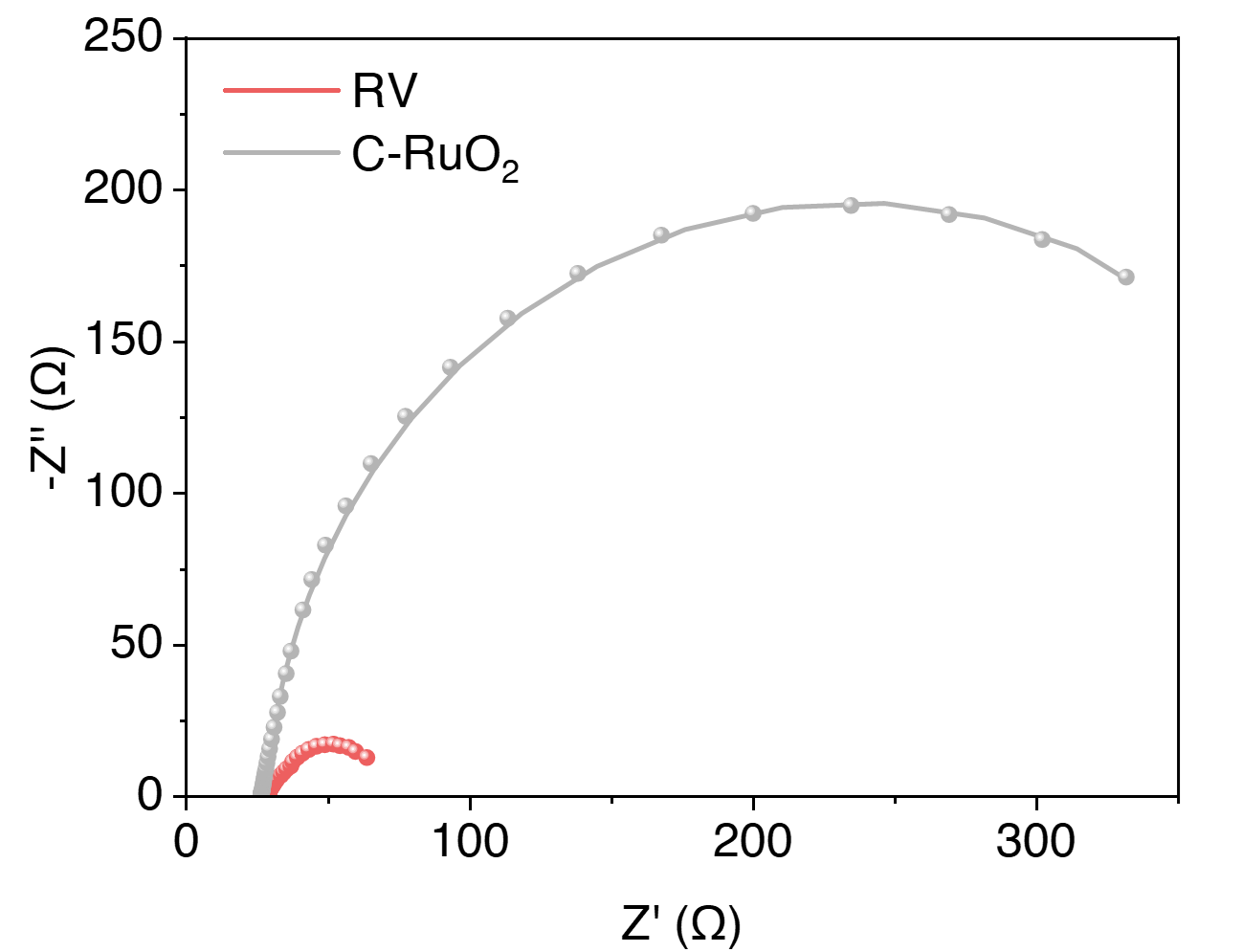


**Figure S5**. Nyquist plots of RV and C-RuO_2_ catalysts.


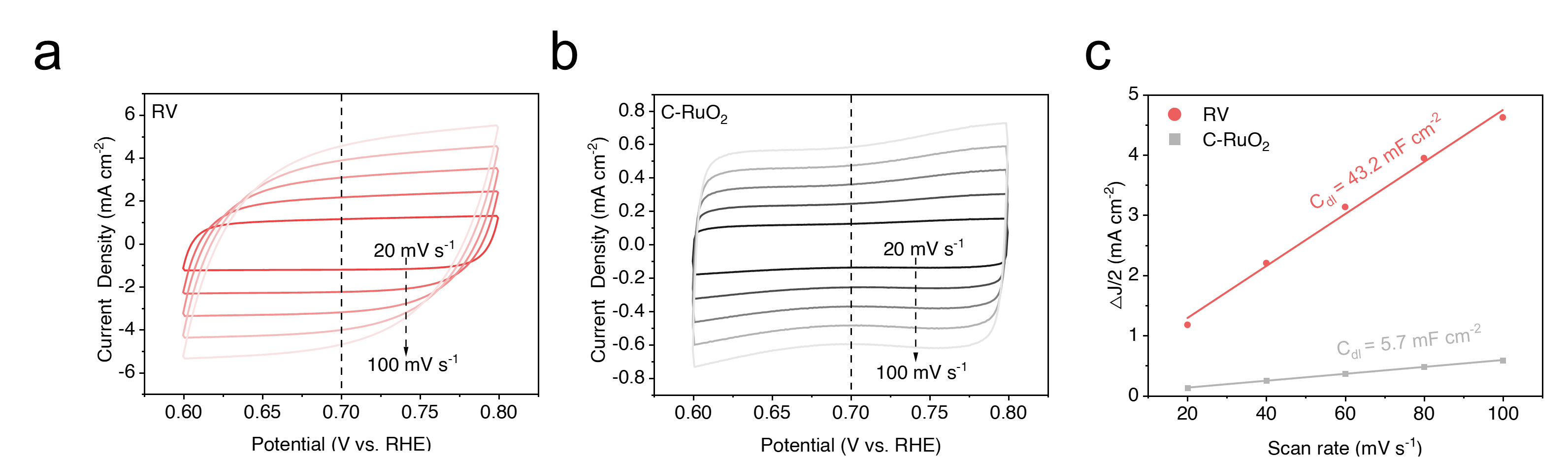


**Figure S6**. CV curves between 0.6 and 0.8 V vs. Ag/AgCl of (a) RV and (b) C-RuO_2_. (c) C_dl_ fitting of RV and C-RuO_2_ catalysts.


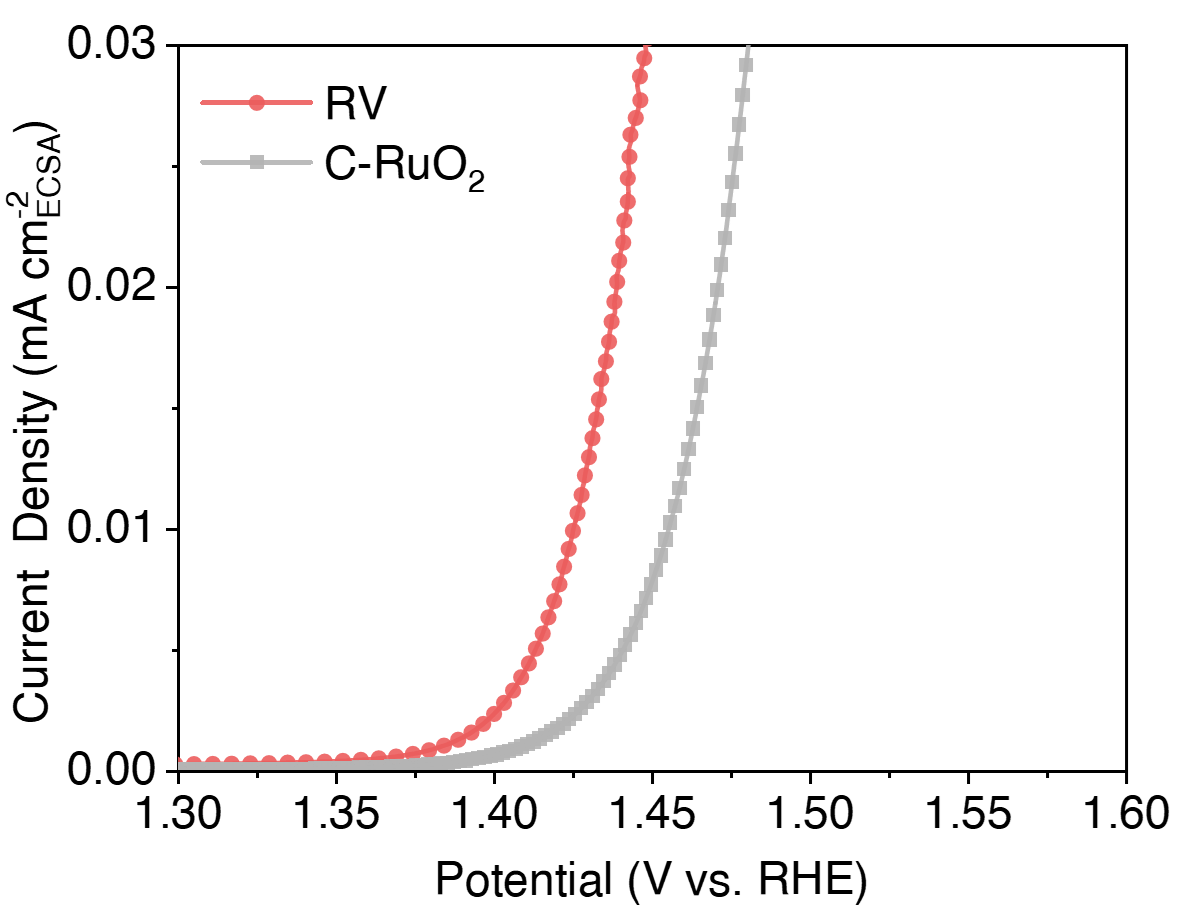


**Figure S7**. ECSA-normalized LSV curves of RV and C-RuO_2_ catalysts.


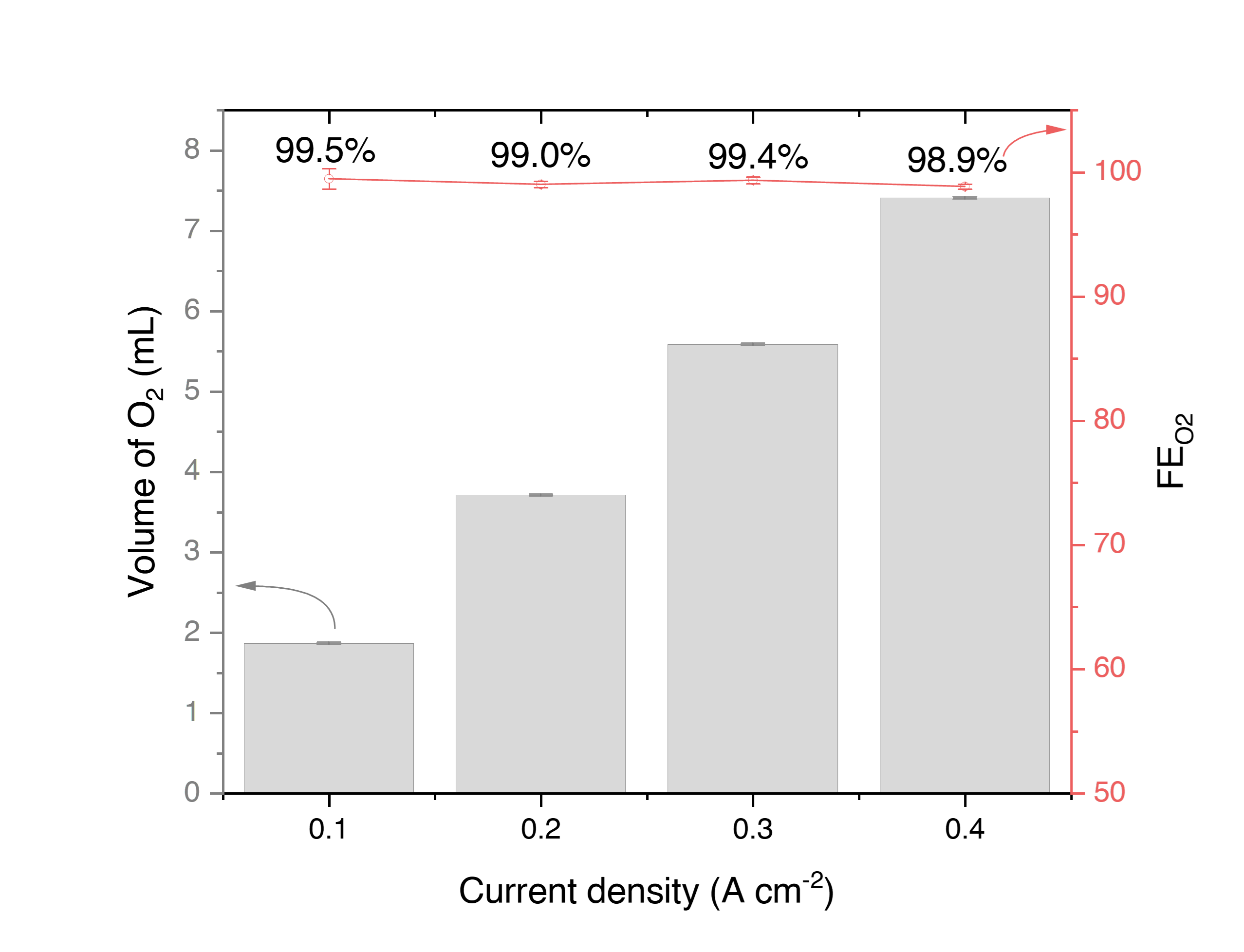


**Figure S8**. O_2_ Faradaic efficiency measured by the water displacement method at current densities of 0.1, 0.2, 0.3, and 0.4 A cm^-2^. The amount of evolved O_2_ was collected over 5 min for calculation. Each measurement was repeated three times.


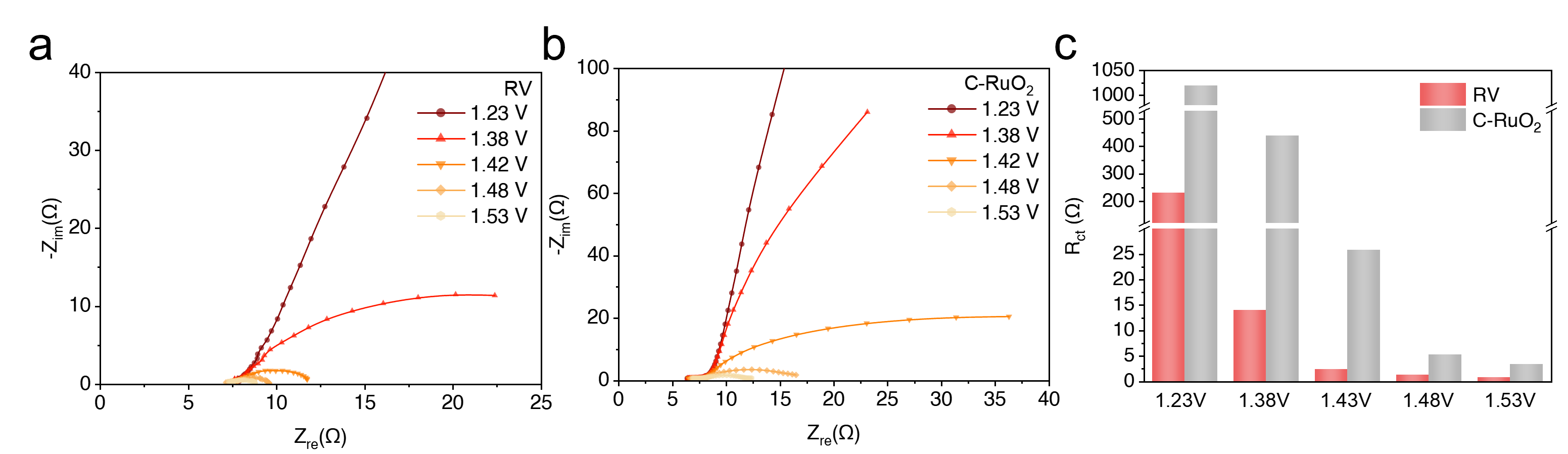


**Figure S9**. Nyquist plots of (a) RV and (b) C-RuO_2_ derived from *in-situ* EIS measurements. (c) Comparison of R_ct_ values of RV and C-RuO_2_ catalysts at different applied potentials.


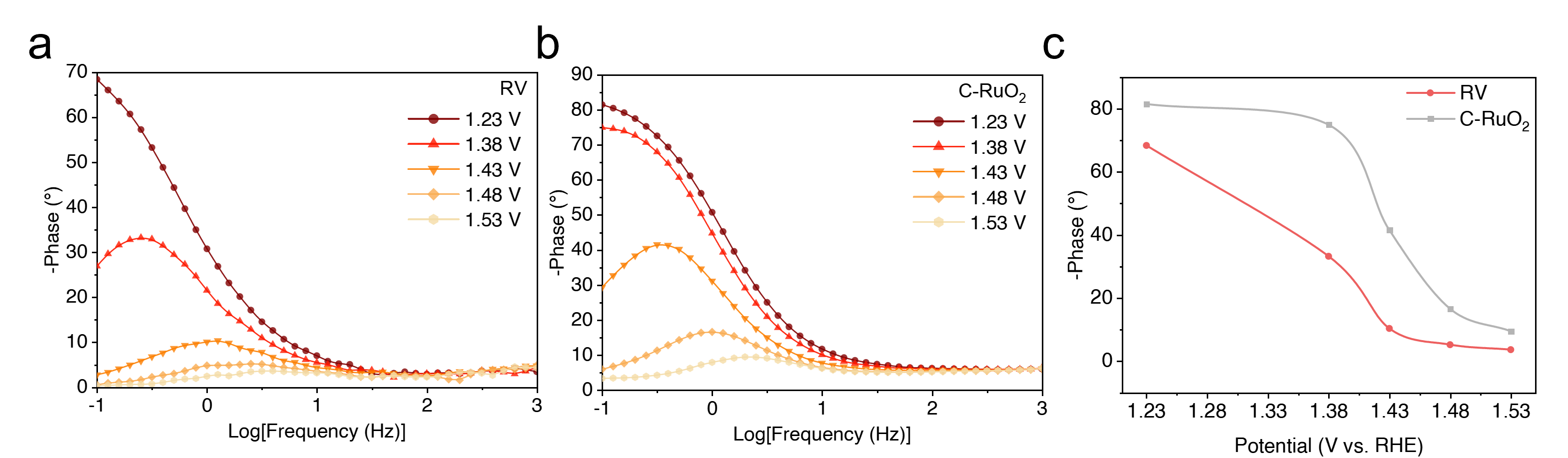


**Figure S10**. Bode phase plots of (a) RV and (b) C-RuO_2_. (c) Response of the phase angle to the applied potential extracted from b) and c).


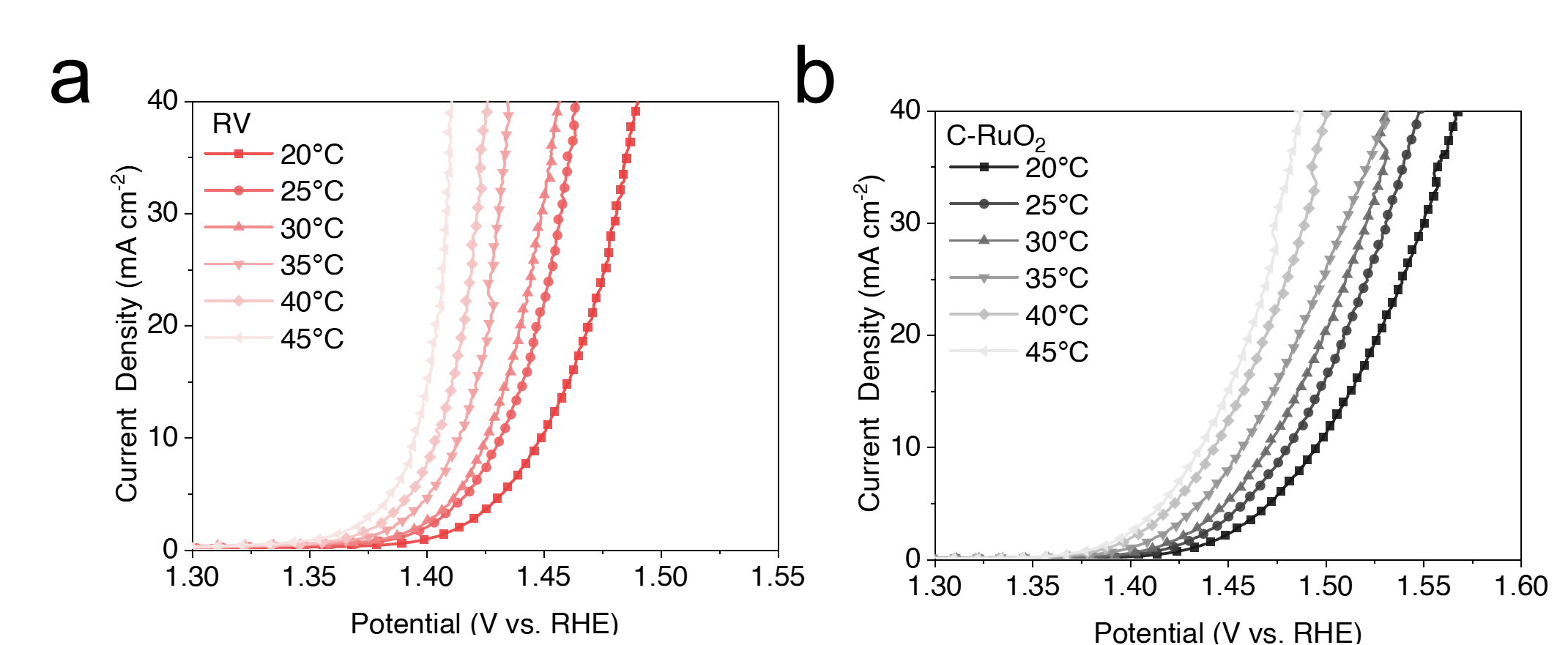


**Figure S11**. LSV curves at different temperatures of (a) RV and (b) C-RuO_2_.


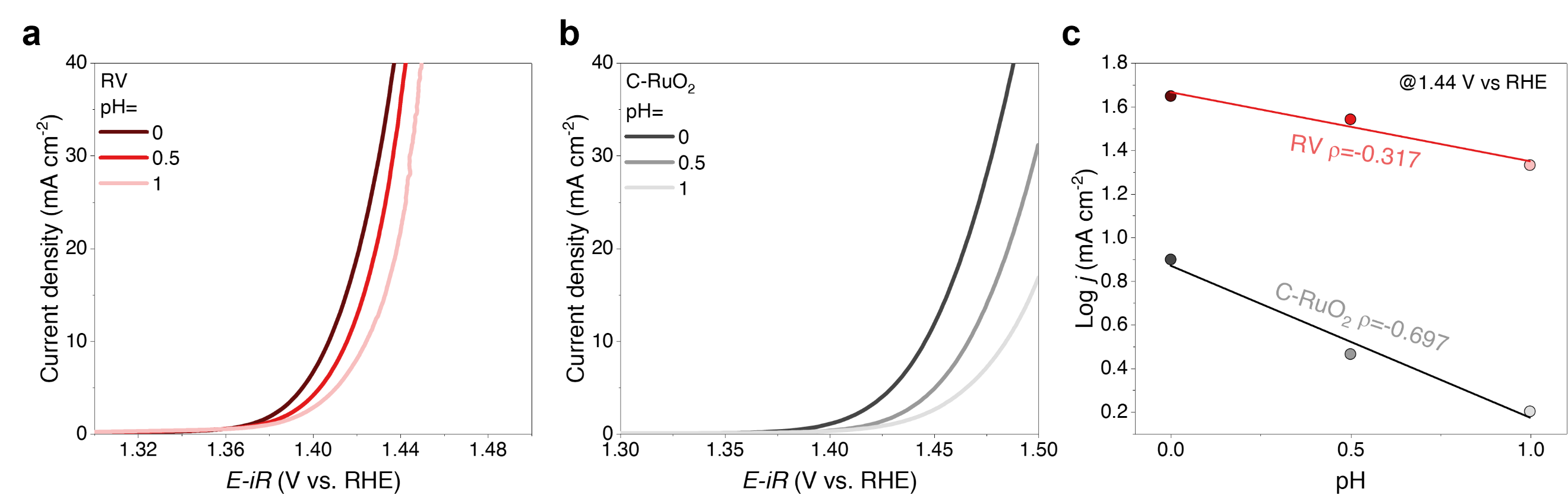


**Figure S12**. LSV curves at different pH of (a) RV and (b) C-RuO_2_. (c) Linear fitting of the log_10_(current density) at 1.44 V vs. RHE as a function of pH.


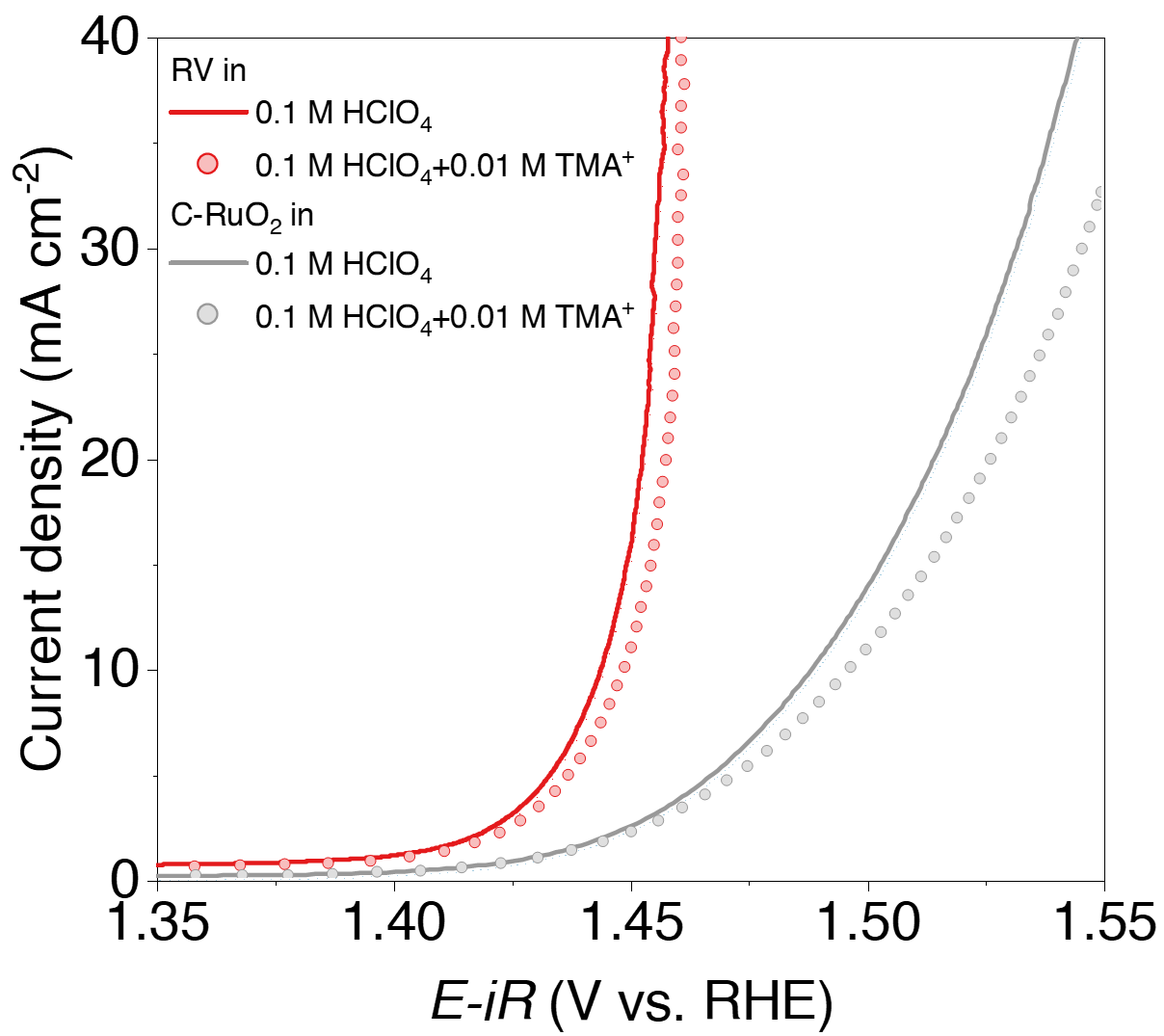


**Figure S13**. LSV curves of RV and C-RuO_2_ measured in 0.1 M HClO_4_ and 0.1 M HClO_4_+0.01 M TMA^+^.


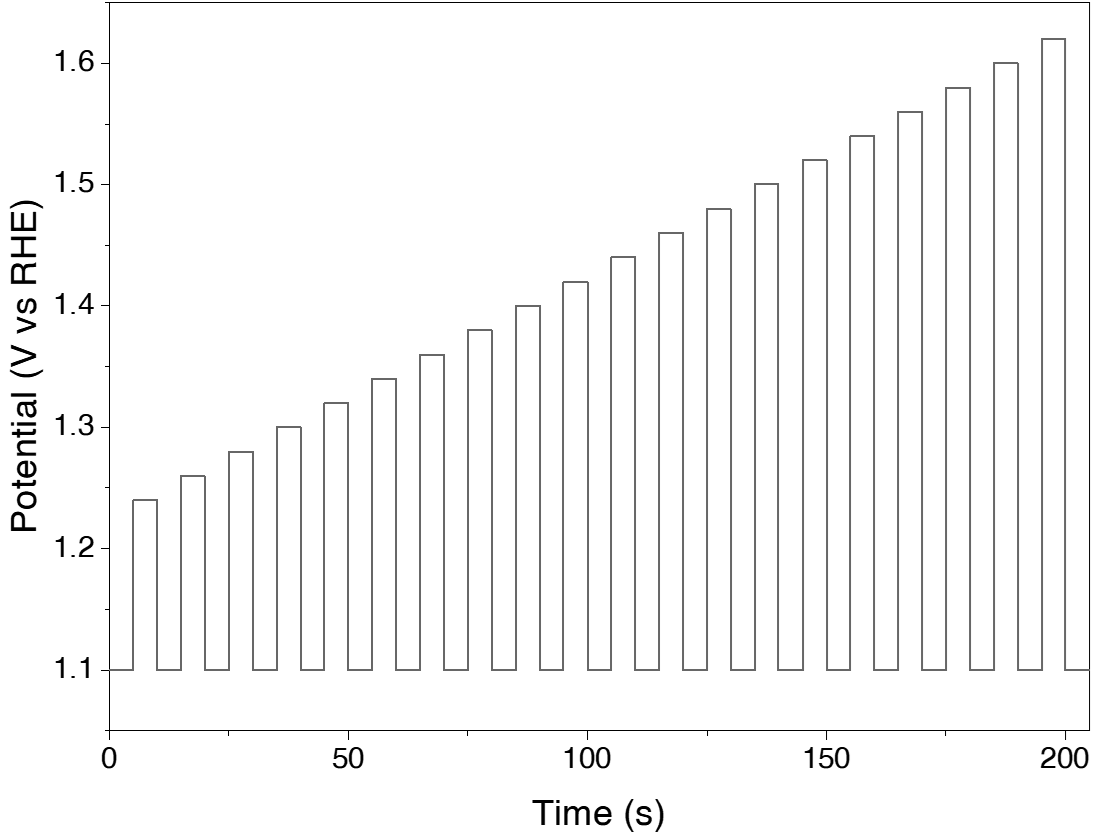


**Figure S14**. Protocol used for PV on RV and C-RuO_2_ catalysts. The measurements were conducted between a cathodic hold potential of 1.10 V vs. RHE and varying anodic non-*i*R corrected potentials ranging from 1.22 V to 1.60 V vs. RHE in 0.1 M HClO_4_ electrolyte.


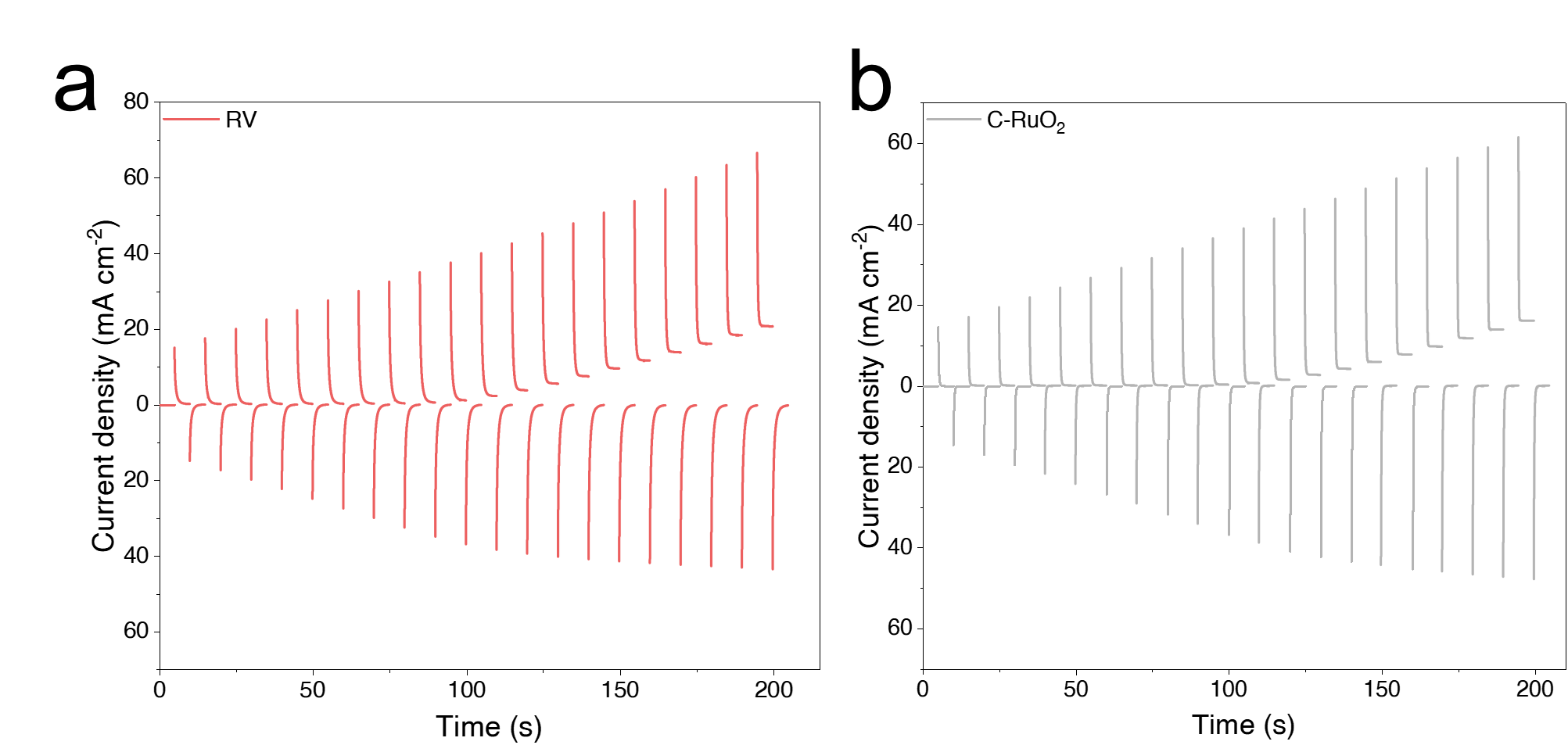


**Figure S15**. Current responses obtained from PV of (a) RV and (b) C-RuO_2_ at various anodic potentials.


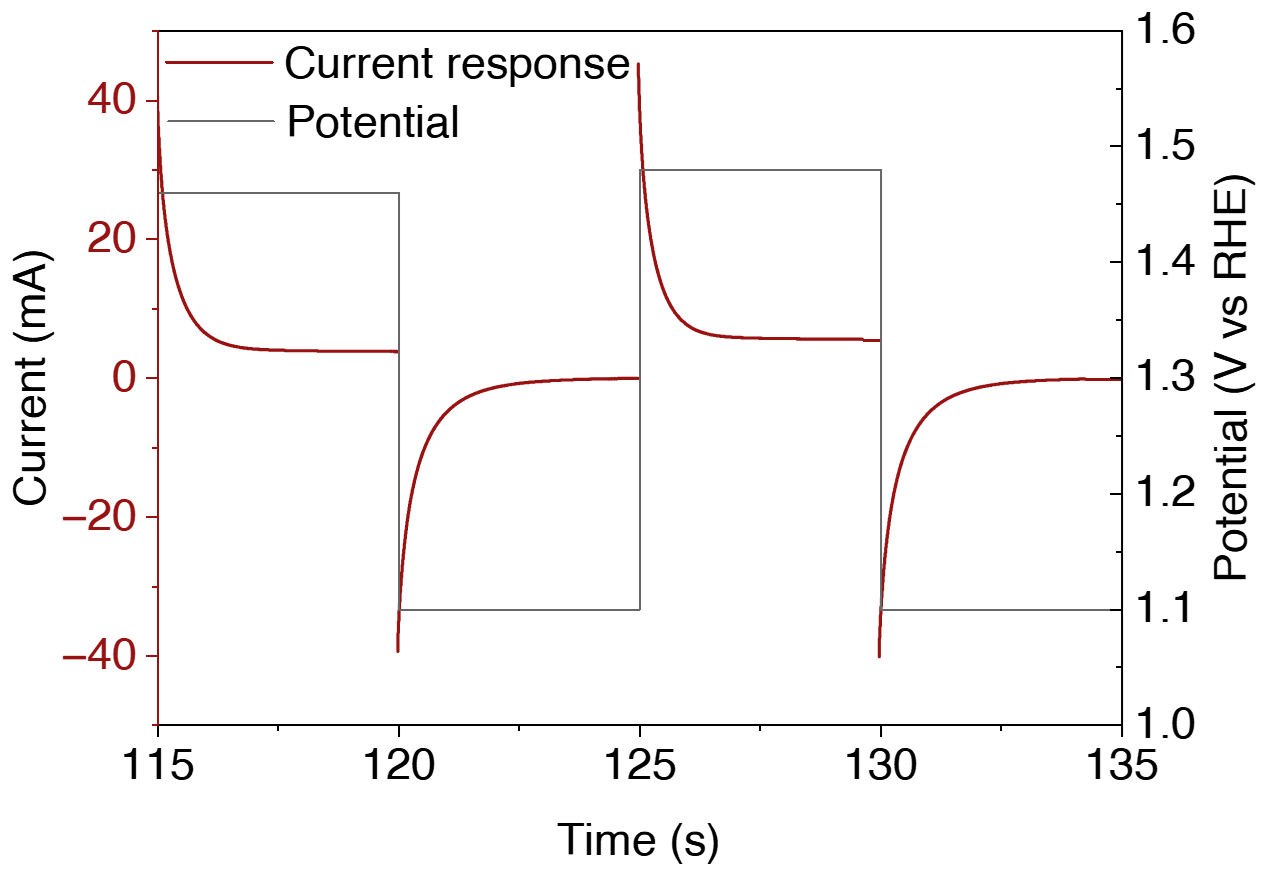


**Figure S16**. Magnified view illustrating the PV protocol cycle and the corresponding anodic/cathodic current responses of RV.


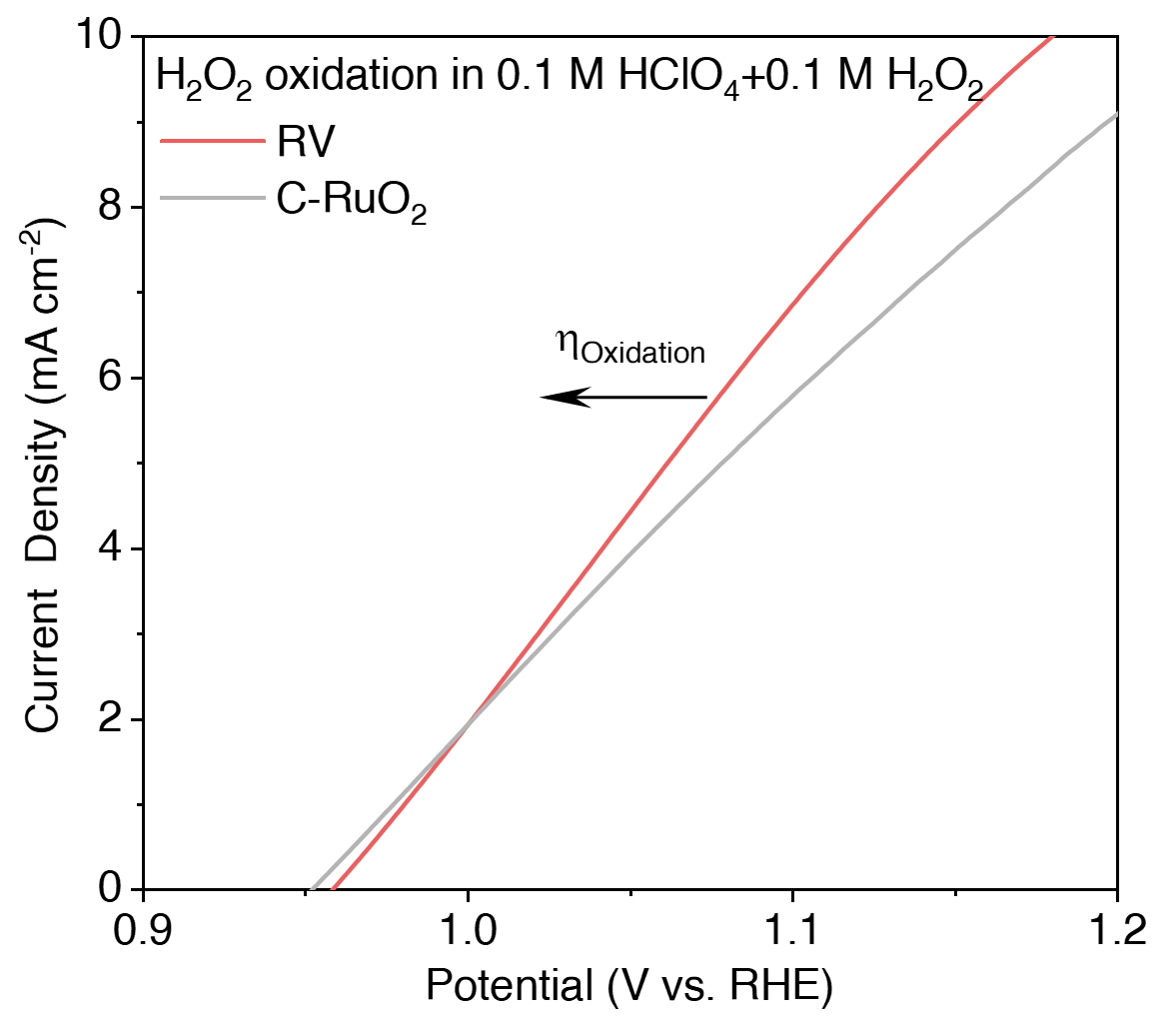


**Figure S17**. LSV curves of RV and C-RuO_2_ in 0.1 M HClO_4_+0.1 M H_2_O_2_.


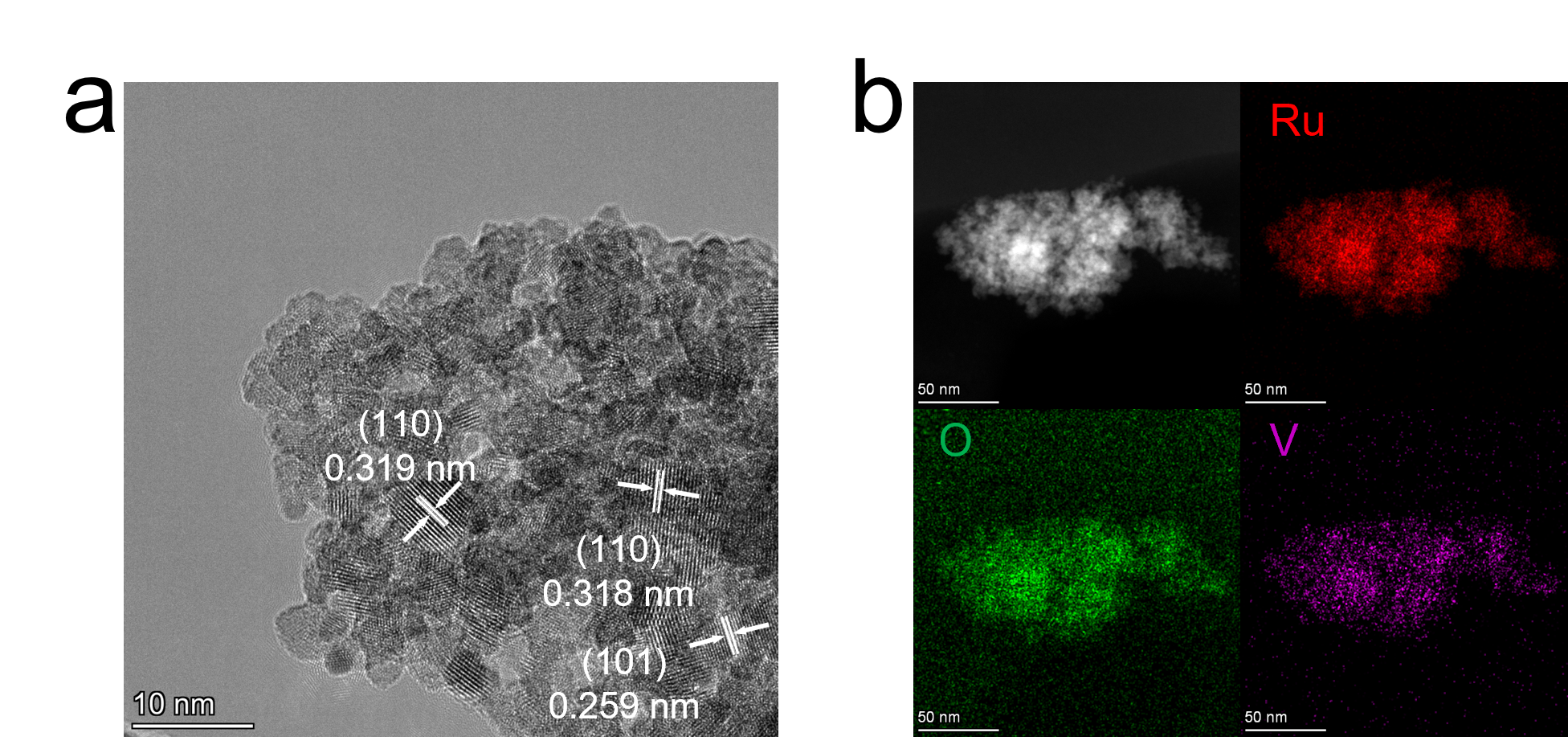


**Figure S18**. (a) HRTEM image of RV-24h sample. (b) HAADF-STEM image and the corresponding EDS elemental mapping of RV-24h sample.


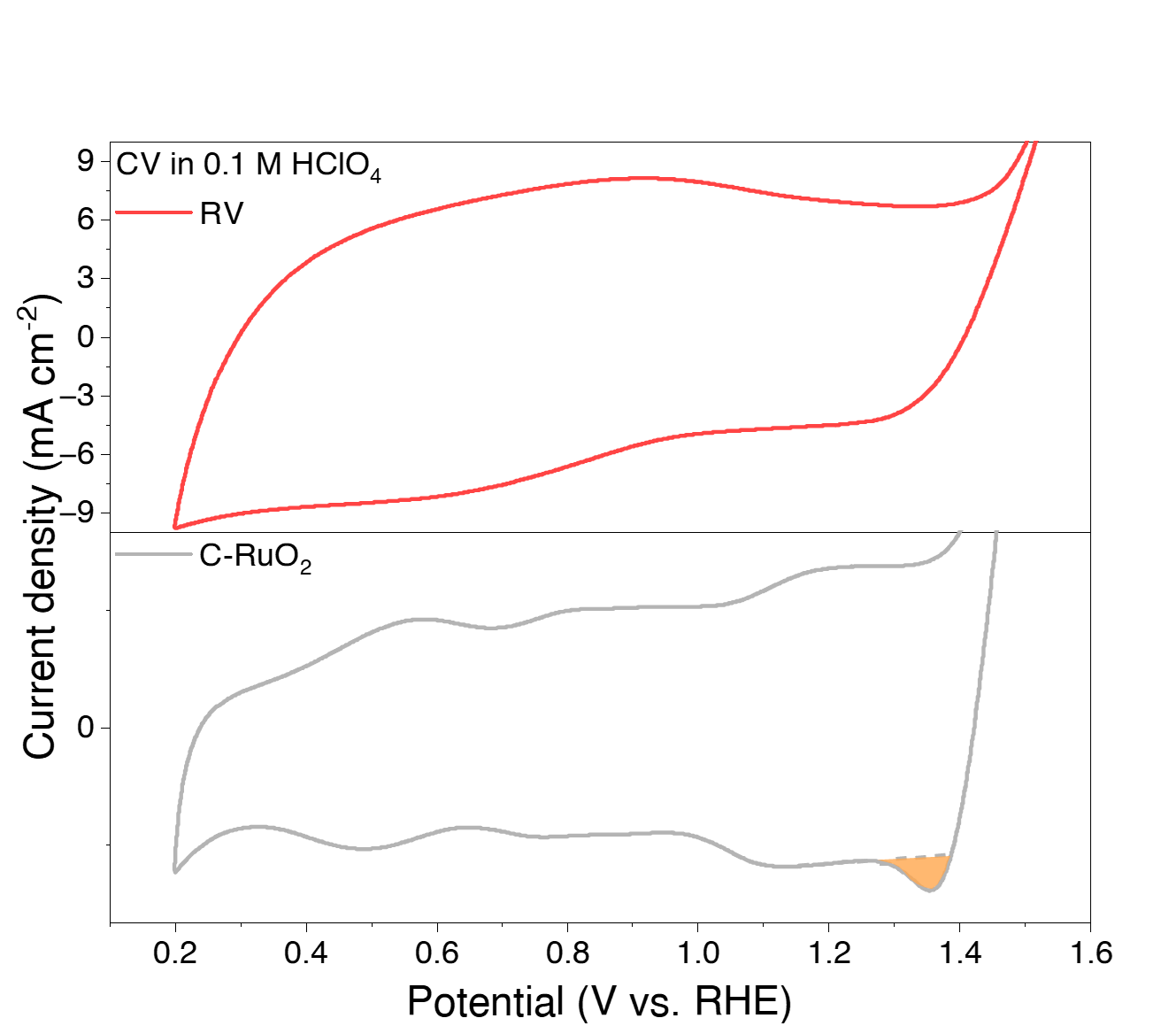


**Figure S19**. CV curves in 0.1 M HClO_4_ of RV and C-RuO_2_.


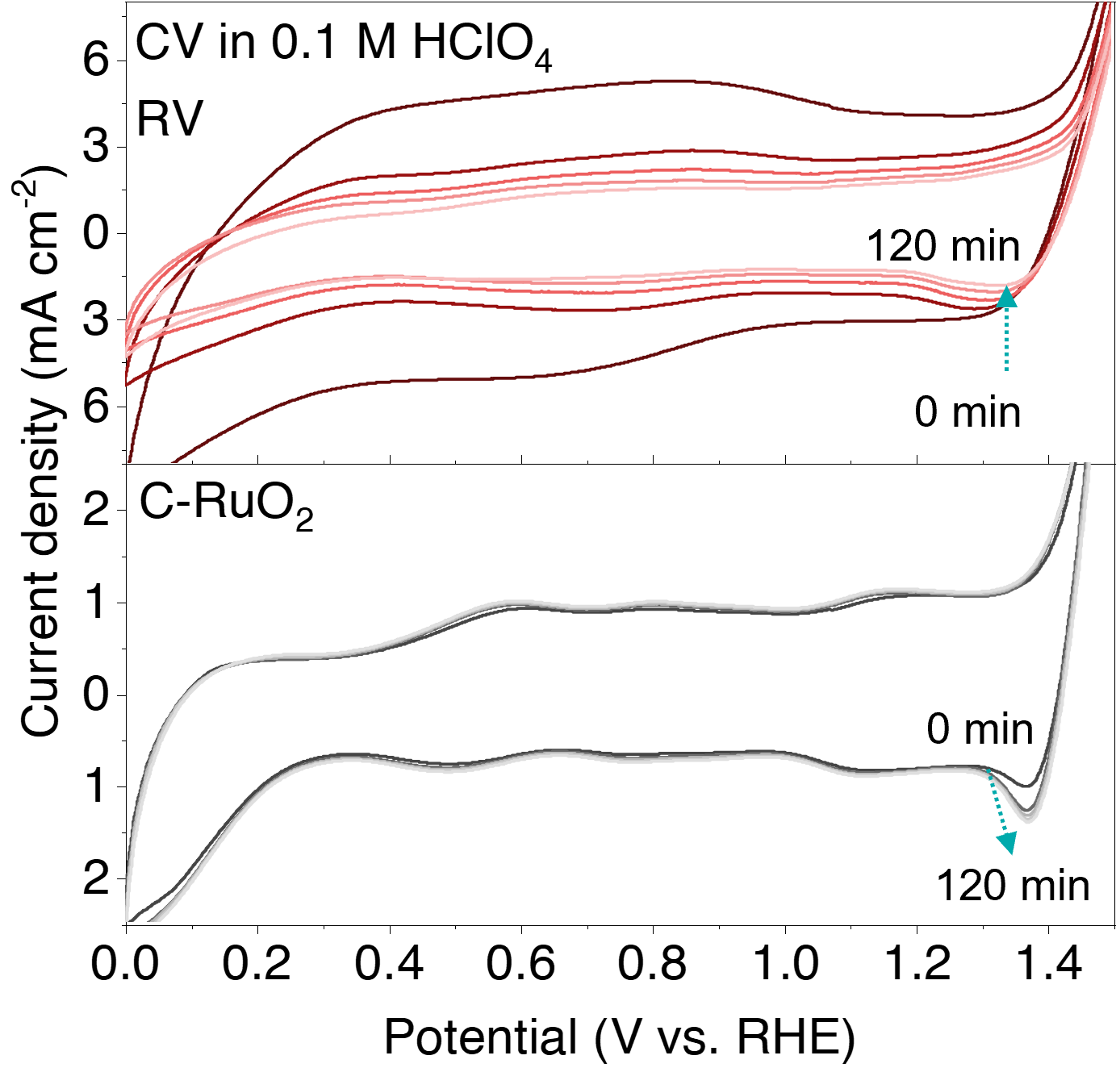


**Figure S20**. CV curves of RV and C-RuO_2_ recorded in 0.1 M HClO_4_ after chronopotentiometric operation at 10 mA cm^-2^ for varying durations (0, 30, 60, 90, and 120 min).


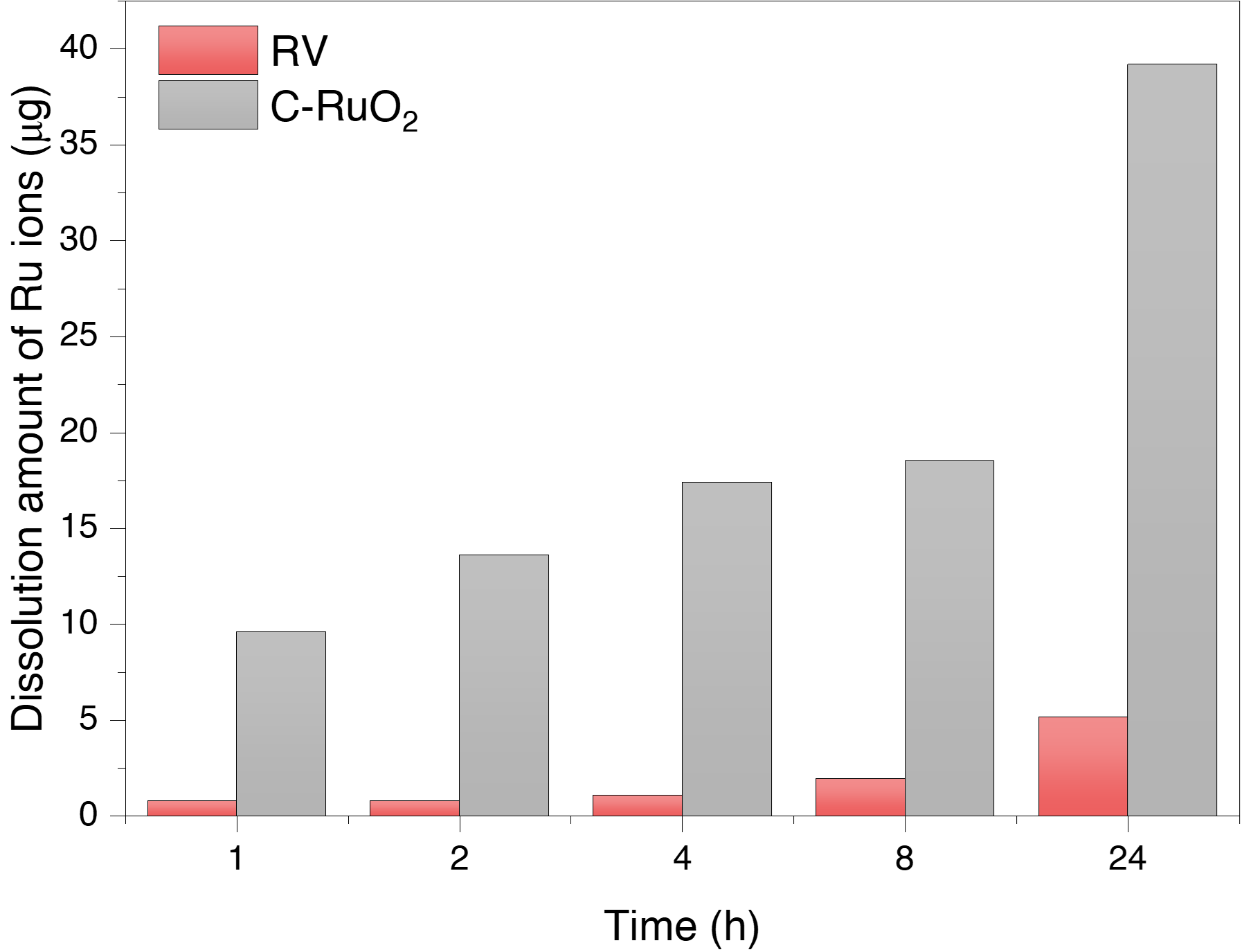


**Figure S21**. Dissolution of Ru ions across different reaction intervals of RV and C-RuO_2_ at 10 mA cm^-2^.

**Table S1**. Summary of the literature-screening workflow, category, sample counts, and descriptive statistics used in the data-mining analysis.

| **Step** | **Group** | **Sample count (n)** | **Median overpotential**  **(mV at 10 mA cm^-2^)** | **Min-max (mV)** | **Notes** |
| --- | --- | --- | --- | --- | --- |
| Literature collection | Reported OER catalysts in last 10 years | 12229 | / | / | Records collected from DigCat |
| Initial screening & exclusion | pH=0-1 | 1689 | / | / | Keep acidic OER catalysts and exclude duplicate or unclear catalyst identity |
| Final dataset | / | 718 | / | / | / |
| Metal family | Ru-based | 292 | 226 | 143-670 | / |
| Metal family | Ir-based | 299 | 274 | 166-770 | / |
| Metal family | Co-based | 53 | 392 | 155-885 | / |
| Metal family | Mn-based | 45 | 460 | 196-940 | / |
| Metal family | Others | 29 | 560 | 191-860 | / |
| Ru-based subset | Metal oxides | 146 | 232 | 150-558 | / |
| Ru-based subset | Heterostructures | 90 | 212 | 143-400 | / |
| Ru-based subset | Metal/alloys | 14 | 201 | 155-346 | / |
| Ru-based subset | SACs | 9 | 209 | 144-297 | / |
| Ru oxide comparison | Monometallic Ru oxides | 67 | 283 | 150-450 | / |
| Ru oxide comparison | Multi-metal Ru oxides | 79 | 210 | 154-558 | / |

**Table S2**. Computed energetics (ΔG_O*_-ΔG_HO*_) of the 20 selected M-doped RuO_2_

| Catalysts | ΔG_O*_-ΔG_HO*_ (eV) |
| --- | --- |
| Co-RuO_2_ | 1.21 |
| Fe-RuO_2_ | 1.86 |
| Ni-RuO_2_ | 1.24 |
| Zn-RuO_2_ | 1.23 |
| Cu-RuO_2_ | 1.15 |
| Mn-RuO_2_ | 1.26 |
| Nb-RuO_2_ | 1.21 |
| Re-RuO_2_ | 1.19 |
| Nd-RuO_2_ | 1.24 |
| Sn-RuO_2_ | 1.23 |
| Al-RuO_2_ | 1.26 |
| Zr-RuO_2_ | 1.29 |
| Ta-RuO_2_ | 1.21 |
| Hf-RuO_2_ | 1.29 |
| Ti-RuO_2_ | 1.18 |
| Bi-RuO_2_ | 1.21 |
| Ga-RuO_2_ | 1.35 |
| Cr-RuO_2_ | 1.30 |
| W-RuO_2_ | 1.38 |
| V-RuO_2_ | 1.54 |

**Table S3**. Elemental composition and chemical-state information obtained from XPS.

| **Sample** | **Chemical formula** | **Ratio of**  **V^3+^ : V^4+^ : V^5+^** | **Ratio of O_h_ : O_l_** | **Position of Ru 3*p*_3/2_ (eV)** |
| --- | --- | --- | --- | --- |
| RV | Ru_0.66_V_0.34_O_x_ | 0.30 : 0.42 : 0.28 | 1.66 | 463.2 |

**Table S4**. OER performance metrics of RV and C-RuO_2_.

| **Sample** | ***η_10_* (mV)** | **Tafel slope (mV dec^-1^)** | **C_dl_**  **(mF cm^-2^)** | **ECSA (cm^2^)** | | **MA**  **(A g_Ru_^-1^)** | | **TOF**  **(s^-1^)** |
| --- | --- | --- | --- | --- | --- | --- | --- | --- |
| RV | 193±1 | 41.9 | 43.2 | 241.9 | 536.3±6.0 | | 0.139±0.002 | |
| C-RuO_2_ | 271±5 | 52.6 | 5.7 | 31.9 | 19.7±1.2 | | 0.0052±0.0003 | |

**Table S5**. Benchmarking of the performance of recently reported Ru-based catalysts.

| Catalysts | Electrolyte | Loading (mg cm^-2^) | Support | iR correction | η^a^  (mV) | Tafel slope  (mV dec^-1^) | Stability^b^  (h) | Stability in PEMWEs  (h) | Voltage at 1 A cm^-2^ in PEMWEs (V) | Reference |
| --- | --- | --- | --- | --- | --- | --- | --- | --- | --- | --- |
| RV | 0.1 M HClO_4_ | 0.3 | RDE | 90% | 192 | 41.9 | 3000 | 140 h@0.2 A cm^-2^ | 1.725 V @ 60°C | This work |
| Ta-RuO_2_ | 0.5 M H_2_SO_4_ | 0.25 | RDE | 90% | 226 | 47.1 | / | 2800 h@1 A cm^-2^ | 1.704 V @ 60°C | *Science*, **2025**, *387*, 48-55. |
| Ta/B-RuO_2_ | 0.5 M H_2_SO_4_ | 0.5 | Carbon paper | / | 170 | 44 | 80 | 120 h@0.2 A cm^-2^ | 1.6 V | *Nat. Commun.*, **2025**, *16*, 6716. |
| Pt-RuO_2_ | 0.5 M H_2_SO_4_ | 0.88 | Glassy carbon | / | 215 | 63.89 | 1500 | 500 h@0.5 A cm^-2^ | 1.567 V @80°C | *Nat. Commun.*, **2025**, *16*, 6217. |
| anh-RuO_2_ | 0.5 M H_2_SO_4_ | / | RDE | / | / | / | 2100 | 550 h@1 A cm^-2^ | ~1.68 V @80°C | *Nat. Commun.*, **2025**, *16*, 801. |
| RuO_2_-HEAE | 0.1 M HClO_4_ | 0.5 | Carbon paper | 90% | 201 | 33.31 | / | 1500 h@1 A cm^-2^ | 1.6 V@80°C | *Nat. Commun.*, **2025**, *16*, 6894. |
| Na-RuO_2_ | 0.1 M HClO_4_ | 1 | Carbon paper | 100% | 200 | 49.7 | 1800 | 80 h@0.2 A cm^-2^ | 1.588 V@80°C | *J. Am. Chem. Soc.*, **2025**, *147*, 10446–10458. |
| V_Cd_-RuO_2_ | 0.1 M HClO_4_ | 0.35 | Carbon paper | 95% | 203 | 47.9 | 900 | 600 h@0.2 A cm^-2^ | 1.83 V@60°C | *Angew. Chem. Int. Ed.*, **2026**, e24664. |
| RZW | 0.1 M HClO_4_ | 0.3 | RDE | 90% | 200 | 37.6 | 4000 | / | / | *Angew. Chem. Int. Ed.,* **2025***, 64,* e202422707*.* |
| Cr_0.1_Sn_0.1_Ru_0.8_O_2_ | 0.5 M H_2_SO_4_ | 0.28 | Glassy carbon | / | 178 | 47.3 | 100 | 500 h@1 A cm^-2^ | 1.6 V@80°C | *Angew. Chem. Int. Ed.*, **2025**, *64*, e202515362. |
| Ru_0.5_Mn_0.5_O_2_ | 0.5 M H_2_SO_4_ | 0.35 | Glassy carbon | / | 166 | 41.8 | 2500 | 600 h@0.2 A cm^-2^ | 1.762 V@60°C | *Energy Environ. Sci.*, **2025**, *18,* 3352–3364*.* |
| Bi-RuO_2_ SAAO | 0.5 M H_2_SO_4_ | 1 | Carbon paper | 90% | 192 | 37.8 | 650 | 24 h@0.2 A cm^-2^ | 1.59 V@60°C | *Adv. Mater.*, **2025**, *37,* 2417777*.* |
| Ur-Se-RuO_x_ | 0.1 M HClO_4_ | 2 | Carbon paper | 100% | 226 | 56.7 | / | 1000 h@0.2 A cm^-2^ | 1.62 V@60°C | *Angew. Chem. Int. Ed.*, **2025**, *64*, e202512848. |
| Er-RuO_x_ | 0.5 M H_2_SO_4_ | 0.5 | Carbon paper | 95% | 200 | 45 | 200 | 200 h@0.2 A cm^-2^ | 1.590 V@80°C | *Nat. Commun.*, **2024**, *15*, 4974. |
| ZnRuO_x_ | 0.5 M H_2_SO_4_ | 0.255 | RDE | 95% | 255 | 48 | 320 | 120 h@0.2 A cm^-2^ | 1.68 V@80°C | *J. Am. Chem. Soc.*, **2024**, *146*, 15515−15524. |
| RuCoO_x_ | 1 M HClO_4_ | 0.0428 | Carbon paper | 100% | 200 | 50.1 | 100 | 10 h@0.1 A cm^-2^ | / | *J. Am. Chem. Soc.*, **2023**, *145*, 17995-18006. |

^a^ overpotentials at 10 mA cm^-2^.

^b^ Stability at 10 mA cm^-2^ in three-electrode system.

**Table S6**. Elemental composition and chemical-state information of RV-Xh (X=1, 6, 12, 24) obtained from XPS.

| **Sample** | **Chemical formula** | **Ratio of**  **V^3+^ : V^4+^ : V^5+^** | **Ratio of O_h_ : O_l_** | **Position of Ru 3*p*_3/2_ (eV)** |
| --- | --- | --- | --- | --- |
| RV-1h | Ru_0.79_V_0.21_O_x_ | 0.34 : 0.38 : 0.28 | 1.81 | 462.9 |
| RV-6h | Ru_0.84_V_0.16_O_x_ | 0.36 : 0.34 : 0.30 | 1.88 | 462.9 |
| RV-12h | Ru_0.88_V_0.12_O_x_ | 0.40 : 0.30 : 0.30 | 1.93 | 462.9 |
| RV-24h | Ru_0.87_V_0.13_O_x_ | 0.41 : 0.29 : 0.30 | 1.98 | 462.9 |

References

[42] Gou, W., S. Zhang, Y. Wang, et al., "Oxygen spillover from RuO_2_ to MoO_3_ enhances activity and durability of RuO_2_ for acidic oxygen evolution" *Energy & Environmental Science* **17**, (2024): 6755.

[43] Hafner, J., "Ab-initio simulations of materials using VASP: Density-functional theory and beyond" *Journal of Computational Chemistry* **29**, (2008): 2044.

[44] Blöchl, P. E., "Projector augmented-wave method" *Physical Review B* **50**, (1994): 17953.

[45] Kohn, W., L. J. Sham, "Self-consistent equations including exchange and correlation effects" *Physical Review* **140**, (1965): A1133.

[46] Perdew, J. P., K. Burke, M. Ernzerhof, "Generalized gradient approximation made simple" *Physical Review Letters* **77**, (1996): 3865.

[47] Hammer, B., L. B. Hansen, J. K. Nørskov, "Improved adsorption energetics within density-functional theory using revised Perdew-Burke-Ernzerhof functionals" *Physical Review B* **59**, (1999): 7413.

[48] Larsen, A. H., J. J. Mortensen, J. Blomqvist, et al., "The atomic simulation environment—a Python library for working with atoms" *Journal of Physics: Condensed Matter* **29**, (2017): 273002.

[49] Hansen, H. A., J. Rossmeisl, J. K. Nørskov, "Surface Pourbaix diagrams and oxygen reduction activity of Pt, Ag and Ni(111) surfaces studied by DFT" *Physical Chemistry Chemical Physics* **10**, (2008): 3722.

[50] Nørskov, J. K., J. Rossmeisl, A. Logadottir, et al., "Origin of the overpotential for oxygen reduction at a fuel-cell cathode" *The Journal of Physical Chemistry B* **108**, (2004): 17886.

[51] Bajdich, M., M. García-Mota, A. Vojvodic, J. K. Nørskov, A. T. Bell, "Theoretical investigation of the activity of cobalt oxides for the electrochemical oxidation of water" *Journal of the American Chemical Society* **135**, (2013): 13521.

Author Contributions

Z. L. L., H. L., H. H. L., and C. Z. L. conceived the idea and designed the experiments. Z. L. L., H. L., H. L., and H. H. L. co-wrote the manuscript. H. L., H. H. L., and C. Z. L. supervised the project. Z. L. L. and K. Z. carried out the experiments and analyzed the results. H. L. and H. L. performed DFT calculations and analyzed the results. M.M.L. performed the *in-situ* FTIR experiments. T.R.X., J.Z., Y.T.S., and J.L.C. helped with material characterizations. All authors discussed the results during manuscript preparation. †These authors contributed equally.
